# Optimization of flavonoids extraction and elucidation of antioxidant mechanisms in *Dendrobium flexicaule* using metabolomics and machine learning

**DOI:** 10.1016/j.ultsonch.2026.107950

**Published:** 2026-07-05

**Authors:** Liu Yang, Yuhang Yi, Abdulaziz Nuhu Jibril, Jing Wen, Xing Song, Chenghao Lv, Si Qin

**Affiliations:** a Laboratory of Food Function and Nutrigenomics, College of Food Science and Technology, Hunan Agricultural University, Changsha 410128, China; b Institute of Integrative Medicine, Hunan Provincial Key Laboratory of Liver Visceral Manifestation in Traditional Chinese Medicine, Department of Integrated Traditional Chinese and Western Medicine, Xiangya Hospital, Central South University, Changsha 410008, China; c College of Bioscience and Biotechnology, Hunan Agricultural University, Changsha, Hunan 410128, China; d College of Engineering, Bayero University Kano 700241, Nigeria

**Keywords:** *Dendrobium flexicaule*, Flavonoids, Optimization of extraction process, Machine learning, Antioxidant activity, Nrf2 signaling pathway

## Abstract

Recent studies have demonstrated that flavonoids constitute a major class of bioactive compounds in Dendrobium species, contributing significantly to their pharmacological properties. However, the underutilization of flavonoids from Dendrobium is largely attributable to two interrelated bottlenecks: (1) the absence of systematic phytochemical screening to identify high-flavonoid germplasm resources, and (2) the lack of robust, scalable extraction protocols optimized for both yield and reproducibility. To address these limitations, this study first employed untargeted metabolomics to comparatively characterize the flavonoid profiles across four representative Dendrobium species. Subsequently, we developed an integrated optimization framework combining single-factor experimental screening, response surface methodology (RSM), and machine learning–based predictive modeling to rationally design and validate an efficient, high-yield flavonoid extraction protocol. Results revealed that Dendrobium flexicaule exhibited the highest total flavonoid content among the four investigated species. Under the optimized extraction conditions, 94 % (v/v) ethanol, 68 min extraction time, a material-to-liquid ratio of 1:50 (w/v), and 72 °C, the flavonoid yield reached 8.90 ± 0.17 mg/g dry weight. Among the machine learning models evaluated, the support vector regression (SVR) model demonstrated the strongest predictive accuracy, achieving an R^2^ of 0.9893, an RMSE of 0.082 mg/g, and an MAE of 0.058 mg/g. However, untargeted metabolomic profiling of the optimized extract identified 34 flavonoids, with rutin as the most abundant compound, followed by eriodictyol and naringenin chalcone, both structurally confirmed by reference standards and spectral data. In vitro functional assays demonstrated that the extract exhibited dose-dependent antioxidant activity and significantly alleviated t-BHP induced oxidative stress in HepG2, mechanistically through activation of the Nrf2/ARE signaling pathway. Collectively, this study identifies Dendrobium flexicaule as a high-potential, flavonoid-rich botanical resource and establishes a robust, integrated framework combining untargeted metabolomics with machine learning–driven optimization to enhance both the efficiency of flavonoid extraction and the rigor of downstream bioactivity assessment.

## Highlights

• First integration of metabolomics, RSM and SVR for *Dendrobium flexicaule* (DF) flavonoids extraction.
• 34 flavonoids were identified and rutin was the most abundant in DF.
• Flavonoids of DF attenuated excess oxidative stress via activation of Nrf2/HO-1/NQO1 pathway.

## Graphical abstract

**Figure:**
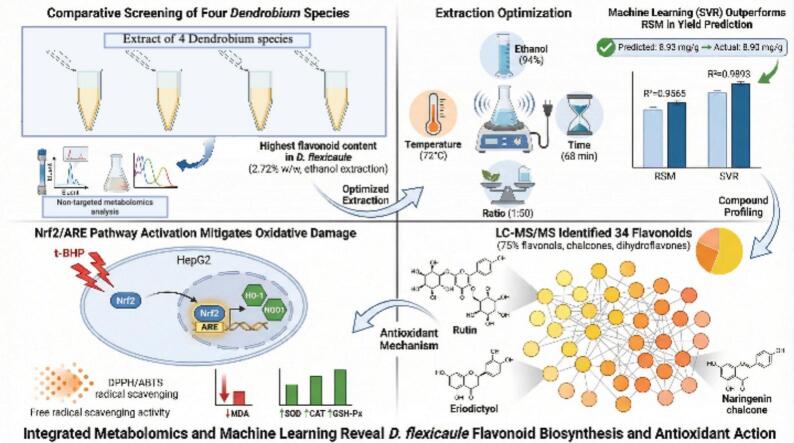

## 1 Introduction

*Dendrobium flexicaule* is a rare and endangered medicinal orchid native to China, traditionally employed in classical Chinese medicine for nourishing yin, clearing heat, and strengthening the stomach [1]. Modern pharmacological studies demonstrate that its primary bioactive constituents are flavonoids, polyphenolic compounds well documented for their potent antioxidant activity [2]. Oxidative stress denotes a pathological imbalance between reactive oxygen species (ROS) generation and the cellular antioxidant defense capacity, resulting in lipid peroxidation, protein oxidation, and DNA damage processes collectively implicated in the pathogenesis of numerous chronic diseases [3]. Flavonoids isolated from Dendrobium officinale mitigate oxidative stress–induced cellular injury through dual mechanisms: direct scavenging of excess reactive oxygen species (ROS) and activation of cytoprotective signaling pathways, including the nuclear factor erythroid 2-related factor 2 (Nrf2) pathway [4].

Recent studies have demonstrated that Dendrobium flexicaule is rich in multiple classes of bioactive compounds, including flavonoids, polysaccharides, and alkaloids, conferring promising potential for applications in functional foods and pharmaceutical development. For example, Li et al. [2] performed a comparative untargeted metabolomic analysis of wild versus cultivated Dendrobium flexicaule and identified 840 metabolites including 91 flavonoids with statistically significant differences in overall metabolite profiles between the two sources. Yuan et al. [5] performed an integrated multi-omics analysis across Dendrobium officinale, Dendrobium huoshanense, and Dendrobium flexicaule, identifying 644 differentially accumulated metabolites revealing species-specific flavonoid accumulation patterns among the three taxa.

Oxidative stress is mechanistically implicated in the onset and progression of numerous chronic diseases. Natural antioxidants particularly flavonoids play a critical role in restoring redox homeostasis through direct free radical scavenging and modulation of endogenous antioxidant systems [6]. Their antioxidant activity is mechanistically linked to activation of the nuclear factor erythroid 2-related factor 2 (Nrf2) signaling pathway and consequent upregulation of downstream cytoprotective enzymes including heme oxygenase-1 (HO-1) and NAD(P)H quinone dehydrogenase 1 (NQO1) [7], [8]. Recent studies have demonstrated that flavonoids isolated from Dendrobium species confer antioxidant protection primarily through activation of the nuclear factor erythroid 2-related factor 2 (Nrf2) signaling pathway. Zhu et al.[4] identified 20 flavonoids from Dendrobium officinale flowers and experimentally validated their antioxidant activity through activation of the PI3K/Akt/Nrf2 signaling axis. Li et al. [9] demonstrated that microbial fermentation significantly enhances both the bioaccessibility and antioxidant activity of flavonoids isolated from *Dendrobium officinale,* as validated by an in vitro gastrointestinal digestion model. Ren et al.[10] revealed that a *Dendrobium officinale* extract alleviates aging-associated kidney injury via activation of the PI3K/Akt/Nrf2/HO-1 signaling axis, as supported by integrated network pharmacology prediction and experimental validation. These findings provide a mechanistic foundation for investigating how flavonoids from Dendrobium flexicaule modulate oxidative stress.

Response surface methodology (RSM) and machine learning (ML) approaches have been increasingly applied in recent food science research to optimize complex experimental processes and predict bioactive compound yields. [11]. RSM is a statistical and mathematical technique that models the quantitative relationship between a set of controllable experimental factors and their corresponding response variables [12]. Machine learning (ML) models have been increasingly adopted for modeling and predicting natural product extraction processes, owing to their demonstrated capacity to handle limited sample sizes, intrinsic nonlinearity, and complex multivariate interactions [13]. Empirical studies demonstrate that machine learning algorithms, including support vector regression (SVR) and artificial neural networks (ANNs) achieve higher prediction accuracy than traditional response surface methodology (RSM) in modeling natural product extraction processes [14].

These findings provide a robust theoretical framework and mechanistic basis for the utilization of *Dendrobium flexicaule* in functional foods and pharmaceutical applications. Although flavonoids from *D. flexicaule* exhibit promising bioactivities, efficient extraction remains a critical prerequisite for their chemical characterization, biological evaluation, and industrial application. Response surface methodology (RSM) has been widely employed for extraction optimization because of its ability to evaluate the effects and interactions of multiple process variables while reducing experimental workload. Nevertheless, RSM relies on polynomial regression models and may be limited in describing the high-order nonlinear relationships and complex multivariate interactions frequently encountered in natural product extraction systems [15]. Recently, machine learning (ML) algorithms, particularly artificial neural networks (ANNs) and support vector regression (SVR), have demonstrated superior predictive performance by effectively capturing nonlinear patterns and multidimensional interactions, even when trained on relatively small datasets [13].

Despite the advances in integrating machine learning models into natural food process engineering, studies focusing on *Dendrobium* species have predominantly employed single optimization approaches for the extraction process. For instance, Shao et al. (2021) [16] optimized flavonoid extraction from *Dendrobium officinale* flowers using deep eutectic solvents combined with RSM, while Han et al. (2025) [17] employed an orthogonal experimental design to improve polysaccharide extraction from *Dendrobium huoshanense* leaves. Additionally, the synergistic integration of response surface methodology (RSM) and machine learning (ML) for optimizing flavonoid extraction from Dendrobium flexicaule remains unexplored. To address these limitations, our study integrated the untargeted metabolomics profiling, multi-factor RSM optimization, and machine learning–driven predictive modeling with support vector regression (SVR) specifically for flavonoid extraction from Dendrobium flexicaule. To the best of our knowledge, no prior study has incorporated these complementary approaches for this species.

Therefore, this study aims to (1) systematically optimized ultrasound-assisted extraction (UAE) parameters for flavonoids from Dendrobium flexicaule (FDF) through synergistic integration of response surface methodology (RSM) and machine learning (ML) regression models followed by rigorous comparative evaluation of their predictive accuracy; (2) comprehensively characterized the chemical profile of the RSM-ML-optimized flavonoid extract using UHPLC-QTOF-MS/MS; (3) evaluated the in vitro antioxidant capacity via standardized assays including DPPH and ABTS^+^ radical scavenging activities and ferric reducing antioxidant power (FRAP); and (4) investigated the cytoprotective efficacy against t-BHP induced oxidative stress in HepG2 cells, with mechanistic focus on Nrf2 nuclear translocation and downstream HO-1 expression. This integrated strategy establishes a transferable framework for optimizing bioactive compound extraction and substantiates the therapeutic potential of D. flexicaule flavonoids as Nrf2-mediated antioxidant agents.

## 2 Materials and methods

### 2.1 Materials and reagents

*Dendrobium flexicaule* was purchased from a plantation in Chongqing, China. *Dendrobium huoshanense, Dendrobium officinale* 3-year-old, and *Dendrobium officinale* 5-year-old were purchased from plantations in Hunan. Absolute ethanol, Yongda reagent, Sodium hydroxide, aluminum nitrate, potassium persulfate, and sodium fluorescein were purchased from Shanghai McLean Chemical Reagent Factory. Rutin standard, 2,2-azobis (2-methylpropylimide) dihydrochloride, 1,1-Diphenyl-2-picrylhydrazyl radical (DPPH), 2,2-diazo-bis (3-ethyl-benzthiazoline-6-sulfonic acid) diammonium salt (2,2′-azino-bis- (3-ethylbenzthiazoline-6-sulfonic acid), and ABTS were obtained from Shanghai Yuanye Biotechnology Co., Ltd. Vitamin C was purchased from Aladdin. The HepG2 cell line was preserved in the laboratory. MEM medium, penicillin/streptomycin, trypsin-EDTA (0.25 %), and protein molecular weight standard were obtained from Thermo Fisher Scientific (USA). PBS buffer was purchased from GIBCO, USA. Fetal bovine serum was obtained from Shanghai Xiaopeng Biotechnology Co., Ltd. Dimethyl sulfoxide, Cell Counting Kit-8 (CCK-8) solution, Cell lysates, and 5 × protein loading buffer were purchased from Shanghai Biyuntian Biotechnology Co., Ltd. BCA protein concentration determination kit, Biosharp Canada; PVDF membrane, TEMED, Tris base, acrylamide were obtained from Beijing Solebao Technology Co., Ltd. Tween-20 was purchased from AMRESCO, and Methanol was obtained from Sinopharm Chemical Reagent Co., Ltd. Skim milk powder was obtained from Yili Group Co., Ltd. Chromogenic solution from NCM Xinsamei. The primary antibodies against NQO1, HO-1, Keap1, and Nrf2 were purchased from Cell Signaling Technology, and secondary antibodies (Anti-Mouse and Anti-Rabbit) from Proteintech. All reagents were analytical grade.

### 2.2 Raw material pretreatment

The raw materials were pretreated with reference to the method provided by Wu et al. [18] with slight modifications. Samples of *Dendrobium flexicaule*, *Dendrobium huoshanense, Dendrobium officinale* 3-year-old, and *Dendrobium officinale* 5-year-old were crushed through a high-speed grinder, passed through a 60-mesh sieve, and stored in a cool, dry, dark place. Approximately 0.05 g of *Dendrobium flexicaule* powder was weighed and extracted with ethanol under specified conditions (material-to-liquid ratio, ultrasonic temperature, extraction time, and ultrasonic power of 300 W). The extract was centrifuged at 12,000 rpm for 10 min, and the supernatant was collected and adjusted to 2.5 mL with 80 % ethanol.

### 2.3 Non-targeted metabolomics analysis

#### 2.3.1 Sample preparation and LC-MS/MS acquisition

Samples were lyophilized using a freeze dryer (Scientz-100F) at − 50 °C and 0.1 mbar for 63 h, followed by homogenization to a fine powder in a mixer mill (MM 400, Retsch) at 30 Hz for 1.5 min. An accurately weighed aliquot of 50 mg of the powdered sample was extracted with 1200 μL of ice-cold 70 % aqueous methanol containing an internal standard (250 μg/mL, prepared by dissolving 1 mg of pure standard in 1 mL of 70 % methanol). The extraction mixture was vortexed six times (30 s per cycle, with 30-min intervals between cycles) to ensure thorough analyte release, centrifuged at 12,000 × g for 3 min at 4 °C, and the resulting supernatant was filtered through a 0.22 μm polytetrafluoroethylene (PTFE) membrane before UPLC-MS/MS analysis.

Chromatographic separation was achieved on a Waters ACQUITY Premier UPLC HSS T3 column (1.8 μm, 2.1 × 100 mm) maintained at 40 °C with a flow rate of 0.4 mL/min. Mobile phase A consisted of 0.1 % (v/v) formic acid in ultrapure water, and mobile phase B consisted of 0.1 % (v/v) formic acid in HPLC-grade acetonitrile. A linear gradient elution program was applied as follows: 0–7.5 min, 5–95 % B; 7.5–8.5 min, isocratic hold at 95 % B; 8.5–18.5 min, re-equilibration to 5 % B. The injection volume was 4 μL.

Mass spectrometric analysis was conducted on an AB Sciex TripleTOF 6600 + system operating in both positive and negative electrospray ionization (ESI) modes. Source parameters were optimized as follows: nebulizer gas (GS1), 50 psi; heater gas (GS2), 50 psi; curtain gas, 25 psi; ion source temperature, 550 °C; declustering potential (DP), ±60 V; and ion spray voltage floating (ISVF), ±5500 V. Full-scan TOF MS spectra were acquired over an *m*/*z* range of 50–1000 with a dwell time of 200 ms. For data-dependent acquisition (DDA), product ion scans were triggered upon precursor detection, using a collision energy (CE) of ± 30 eV (with CE spread of 15 eV), an *m*/*z* range of 25–1000, and a dwell time of 40 ms per scan.

#### 2.3.2 Data processing and identification

Raw LC-MS data files were converted to mzXML format using ProteoWizard (v3.0.20118), followed by peak detection, retention time alignment, and nonlinear retention time correction using XCMS Online (v3.15). Features exhibiting > 50 % missing values across any experimental group were excluded from downstream analysis; remaining missing values were imputed using k-nearest neighbors (KNN) with k = 5. Peak intensities were normalized via support vector regression (SVR)-based batch-effect correction to mitigate instrumental drift. Metabolite identification was conducted through a tiered annotation strategy: (i) matching against an in-house reference library of authenticated flavonoid standards; (ii) spectral matching to public databases (HMDB v4.7, METLIN v5.0); (iii) in silico prediction using CFM-ID 4.0; and (iv) integration with the metDNA 2.0 platform for MS/MS spectral networking. Only features satisfying both criteria comprehensive annotation score ≥ 0.5 (as calculated by metDNA) and relative standard deviation (RSD) of QC sample intensities < 30 % were retained for statistical analysis.

#### 2.3.3 Metabolite identification confidence (non-targeted analysis)

Metabolomics Standards Initiative (MSI) framework as follows: Level 2 (putatively annotated compound) for features matching authentic standards in our in-house reference library acquired under identical chromatographic and MS/MS conditions with a metDNA-derived comprehensive annotation score ≥ 0.5 and QC sample relative standard deviation (RSD) < 30 %; Level 3 (putatively characterized compound class) for features identified solely via spectral matching to public databases (HMDB v4.7, MassBank, MoNA v2023) or in silico prediction using CFM-ID 4.0; and Level 4 (exact mass only) for features assigned a molecular formula based on high-resolution mass accuracy (< 5 ppm error) without structural evidence. In the comparative metabolomic analysis across the four Dendrobium species (Section 3.1), flavonoid classes were evaluated semi-quantitatively using normalized peak intensities without absolute quantification; consequently, all reported class-level comparisons are supported by MSI Level 2 or Level 3 identifications.

### 2.4 Targeted metabolomics analysis for flavonoid quantification

#### 2.4.1 Sample preparation

Accurately weighed aliquots (20 mg) of freeze-dried Dendrobium flexicaule powder were extracted with 500 μL of ice-cold 70 % aqueous methanol containing 10 μL of an internal standard mixture (4000 nmol/L). Extraction was performed by probe sonication at 40 kHz and 120 W for 30 min in an ice-water bath to prevent thermal degradation. Samples were then centrifuged at 12,000 × g for 5 min at 4 °C, and the supernatant was filtered through a 0.22 μm polytetrafluoroethylene (PTFE) membrane. Aliquots of the filtrate were transferred to low-binding glass vials and stored at − 80 °C until LC-MS/MS analysis.

#### 2.4.2 UPLC conditions

Chromatographic separation was performed on a Waters ACQUITY UPLC HSS T3 C18 column (1.8 μm, 100 mm × 2.1 mm). Mobile phase A was 0.05 % formic acid in water, and mobile phase B was 0.05 % formic acid in acetonitrile. The flow rate was 0.35 mL/min, column temperature 40 °C, and injection volume 2 μL. The gradient elution program was: 0 min, 90 % A; 1 min, 80 % A; 9 min, 30 % A; 12.5 min, 5 % A; 13.5 min, 5 % A; 13.6 min, 90 % A; 15 min, 90 % A.

#### 2.4.3 MS/MS conditions and MRM acquisition

Mass spectrometry was performed on a QTRAP 6500 + system (Sciex) equipped with an electrospray ionization source. Source parameters were: ion source temperature 550 °C; ion spray voltage 5500 V (positive mode) and −4500 V (negative mode); curtain gas 35 psi; ion source gas 1 and 2 at 50 psi each. Data acquisition was performed in scheduled multiple reaction monitoring (MRM) mode using Analyst 1.6.3 software. Each MRM transition was optimized for declustering potential (DP) and collision energy (CE). Quantification was performed using MultiQuant 3.0.3 software.

#### 2.4.4 Metabolite identification confidence (MSI Level)

Identification confidence was assigned according to the Metabolomics Standards Initiative (MSI) framework. A total of 34 flavonoids were identified. Among them, 33 compounds were assigned to MSI Level 1 (validated identification) using authentic standards, and one compound (corylin, Flavonoid_104) was assigned to MSI Level 2 (putatively annotated) based on MS/MS spectral matching against public databases (HMDB, MassBank) in the absence of an authentic standard. The detailed list of all 34 flavonoids with their MSI levels, retention times, molecular formulas, and CAS numbers is provided in Supplementary Table S1.

### 2.5 Single-factor experiments

Single factor experiments were carried out to investigate the effects of ultrasonic temperature (30 °C, 40 °C, 50 °C, 60 °C, 70 °C), ultrasonic time (30 min, 60 min, 90 min, 120 min, 150 min), material-to-liquid ratio (1:30, 1:40, 1:50, 1:60, 1:70) and ethanol volume fraction (60 %, 70 %, 80 %, 90 %, 100 %) on the extraction of total flavonoids from *Dendrobium flexicaule*(FDF) under the conditions of material-to-liquid ratio 1:50 (g/mL), ultrasonic time 90 min, ultrasonic temperature 60 °C and ethanol volume fraction 80 %.

### 2.6 Response surface optimization experiment design

The response surface experiment was designed using a four-factor, three-level Box-Behnken design (BBD) using ultrasonic temperature (A), ultrasonic time (B), ethanol volume fraction (C), and material-liquid ratio (D) as independent variables, and the total flavonoid extraction amount (Y) of *Dendrobium flexicaule* as the response variable. Design-Expert software was used to generate the Box-Behnken experimental matrix comprising 29 experimental runs (24 factorial points and 5 center points) to optimize the process parameters of total flavonoids extraction from Dendrobium flexicaule. Factors and levels are shown in Table 1.

**Table 1:** Design factors and levels of response surface experiment.

Level	Factor			
	A (ultrasonic temperature) /°C	B (Extraction time) /min	C (Ethanol volume fraction) /%	D (Material-to-liquid ratio) /mL·g-1)
−1	60	30	80	40
0	70	60	90	50
1	80	90	100	60

**Table 2:** Identification of flavonoids constituents in *Dendrobium flexicaule.*

Number	Metabolite name	Molecular formula	Relative molecular weight	Classification name	Relative content
1	Rutin	C_27_H_30_O_16_	610.15339	Flavonol	29.9902663
2	Quercetin	C_15_H_10_O_7_	302.042655	Flavonol	1.327346873
3	Hyperoside	C_21_H_20_O_12_	464.09548	Flavonol	0.763485639
4	Isorhamnetin	C_16_H_12_O_7_	316.058305	Flavonol	0.604194298
5	Avicularin	C_20_H_18_O_11_	434.084915	Flavonol	0.219686688
6	Isorhamnetin-3-O-glucoside	C_22_H_22_O_12_	478.11113	Flavonol	0.157341028
7	Icariin	C_33_H_40_O_15_	676.236725	Flavonol	0.132741969
8	Kaempferol	C_15_H_10_O_6_	286.04774	Flavonol	0.130223243
9	Quercetin 3-O-sophoroside	C_27_H_30_O_17_	626.148305	Flavonol	0.100006645
10	Astragalin	C_21_H_20_O_11_	448.100565	Flavonol	0.067131003
11	Narcissin	C_28_H_32_O_16_	624.16904	Flavone	3.234056277
12	Apigenin	C_15_H_10_O_5_	270.052825	Flavonoid	0.832946267
13	Kaempferol-3-O-rutinoside	C_27_H_30_O_15_	594.158475	Flavonoid	0.738225739
14	Diosmetin	C_16_H_12_O_6_	300.06339	Flavonoid	0.528288333
15	Luteolin	C_15_H_10_O_6_	286.04774	Flavonoid	0.234544546
16	Apigenin 7-glucoside	C_21_H_20_O_10_	432.10565	Flavonoid	0.085991141
17	Homoplantaginin	C_22_H_22_O_11_	462.116215	Flavone	0.074812194
18	5,7-Dihydroxy-3′,4′,5′-trimethoxyflavone	C_18_H_16_O_7_	344.089605	Flavone	0.03040526
19	Chrysin	C_15_H_10_O_4_	254.05791	Flavone	0.01143245
20	6-Hydroxyflavone	C_15_H_10_O_3_	238.062995	Flavone	0.00657242
21	Naringenin chalcone	C_15_H_12_O_5_	272.068475	Chalcone	8.285198797
22	Trifolin	C_21_H_24_O_10_	436.13695	Chalcone	7.48893148
23	Phloretin	C_15_H_14_O_5_	274.084125	Chalcone	5.436650587

Number	Metabolite name	Molecular formula	Relative molecular weight	Classification name	Relative content
24	Isoliquiritigenin	C_15_H_12_O_4_	256.07356	Chalcone	0.001120048
25	Eriodictyol	C_15_H_12_O_6_	288.06339	Dihydroflavone	20.6555898
26	Isosakuranetin	C_16_H_14_O_5_	286.084125	Dihydroflavone	0.27079379
27	Pinocembrin	C_15_H_12_O_4_	256.07356	Dihydroflavone	0.233785597
28	Taxifolin	C_15_H_12_O_7_	304.058305	Dihydroflavonol	3.611592183
29	Dihydrokaempferol	C_15_H_12_O_6_	288.06339	Dihydroflavonol	1.296219827
30	O-Methylated isoflavone	C_16_H_12_O_5_	284.068475	Isoflavone	0.089289556
31	Psoralidin	C_20_H_16_O_4_	320.10486	Isoflavone	0.059081692
32	Genistein	C_15_H_10_O_5_	270.052825	Isoflavone	0.00666129
33	Vitexin	C_21_H_20_O_10_	432.10565	Flavonoid carbon glycoside	0.02278995
34	Syringaldehyde	C_9_H_10_O_4_	182.05791	Phenolic acids	3.271846527

### 2.7 Machine learning model construction

#### 2.7.1 Dataset and preprocessing

Based on the single-factor experiment and response surface experiment data of FDF, ultrasonic temperature (℃), ultrasonic time (min), ethanol volume fraction (%), and material-liquid ratio (g/mL) were selected as input variables (four features), and the extraction amount of flavonoids (mg/g) was taken as the output response variable. The raw dataset consisted of 29 experimental runs (5 from single-factor experiments and 24 from RSM design matrix), each performed in triplicate, yielding a total of 87 data points after averaging replicates to avoid pseudo-replication. Missing values and obvious outliers were removed prior to modeling. For support vector regression (SVR) models, Z-score normalization was performed on the input variables before modeling using the formula .$x_{\mathrm{normalized}}=\left(x-\mu \right)/\sigma$

where *μ* and *σ* are the mean and standard deviation of each feature.

#### 2.7.2 Data splitting and validation strategy

To prevent data leakage, all replicate measurements under identical process conditions were grouped and assigned to the same fold during cross-validation. The model evaluation adopted a nested cross-validation (CV) framework combined with a grouped 5-fold CV strategy. In the outer loop, the dataset was divided into 5 folds, with four folds used for training and validation (inner loop) and the remaining fold held out for testing. This process was repeated 5 times, with each fold serving as the test set once. The inner loop performed hyperparameter optimization via grid search with another 5-fold CV. The final reported performance metrics are the average over the 5 outer test folds. Random seed was set to 42 for all randomization processes to ensure reproducibility.

#### 2.7.3 Hyperparameter optimization

The following five regression models were implemented and compared: Linear Regression (LR), Support Vector Regression (SVR), Random Forest (RF), Gradient Boosting Decision Tree (GBDT), and XGBoost. The hyperparameter search ranges for each model were as follows:

SVR: kernel = ['linear', 'rbf', 'poly']; C = [0.1, 1, 10, 100]; gamma = ['scale', 'auto', 0.01, 0.1, 1]; epsilon = [0.01, 0.05, 0.1, 0.2].

RF: n_estimators = [50,100,200]; max_depth = [3, 5, 10, None]; min_samples_split = [2], [5], [10]; min_samples_leaf = [1], [2], [4].

GBDT: n_estimators = [50,100,200]; learning_rate = [0.01, 0.05, 0.1, 0.2]; max_depth = [3], [5], [10]; subsample = [0.8, 1.0].

XGBoost: n_estimators = [50,100,200]; learning_rate = [0.01, 0.05, 0.1, 0.2]; max_depth = [3], [5], [10]; subsample = [0.8, 1.0]; colsample_bytree = [0.8, 1.0].

The optimal hyperparameters were selected based on the lowest root mean square error (RMSE) in the inner CV.

#### 2.7.4 Implementation and Hardware

All machine learning models were implemented in Python (version 3.9.16) using the following libraries: scikit-learn (version 1.2.2) for LR, SVR, RF, and GBDT; XGBoost (version 1.7.5) for the XGBoost regressor; and NumPy (version 1.23.5), pandas (version 1.5.3), and Matplotlib (version 3.6.3) for data handling and visualization. All computations were performed on a workstation equipped with an Intel Core i7-12700 K CPU (12 cores, 3.61 GHz) and 32 GB RAM, running the Windows 11 operating system.

#### 2.7.5 Performance metrics

The performance of the model is evaluated by determination coefficient (R^2^), mean absolute error (MAE), and root mean square error (RMSE), and the calculation formula is as follows:

(1) $$R^{2}=1-\sum {\left(i=1\right)}^{n}{\left(y_{i}-\hat{{\left(y\right)}_{i}}\right)}^{2}/\sum {\left(i=1\right)}^{n}{\left(y_{i}-\overset{¯}{\left(y\right)}\right)}^{2}$$

$$MAE=1/n\sum {\left(i=1\right)}^{n}\left|y_{i}-\hat{{\left(y\right)}_{i}}\right|$$

$$RMSE=\sqrt{\left(1/n\sum {\left(i=1\right)}^{n}{\left(y_{i}-\hat{{\left(y\right)}_{i}}\right)}^{2}\right)}$$

Where: $y_{i}$ is Experimental measured values; $\hat{{\left(y\right)}_{i}}$ is Model prediction; $\overset{¯}{\left(y\right)}$ is the mean of measured values; n is the total number of samples.

### 2.8 Purification of FDF

The FDF were extracted according to the response surface optimization conditions, and the FDF was purified according to the method of purifying flavonoids by macroporous resin studied by Xie Y et al.[19] The purified FDF was lyophilized and stored at-20 ℃ for subsequent antioxidant tests. The formula for flavonoid content is as follows: Flavonoid purity/% = ρ total flavonoids × V/m_1_ × 100 (2).

Where: ρ Total flavonoids-for mass concentration calculated according to the rutin standard curve regression equation, g/L；V is the volume of FDF after constant volume, mL; m_1_ is the mass of dried FDF, mg.

### 2.9 Determination of total flavonoids

The total flavonoid content of *Dendrobium flexicaule* samples was determined with reference to the method of Lu et al. [20]which was slightly modified. Briefly, 0.5 mL of extract was mixed with 0.3 mL of 5 % sodium nitrite and incubated for 6 min at room temperature. Then, 0.3 mL of 10 % aluminum nitrate was added and incubated for another 6 min. Finally, 3 mL of 4 % sodium hydroxide solution was added, and the reaction was allowed to stand for 15 min. Absorbance was measured at 510 nm. All tests were conducted in triplicate. The standard curve was determined using rutin as a control: y = 0.3675 x- 0.0026 (R^2^ = 0.999).

### 2.10 Antioxidant activity

#### 2.10.1 DPPH free radical scavenging activity assay

Following the method of Wu et al. [21] with slight modifications. Pipette 50 µL of sample solutions of different concentrations into a 96 −well plate, add 150 µL of LDPPH-methanol working solution, and place in the dark for 30 min. Absorbance was measured at 517 nm in a microplate reader.

#### 2.10.2 Measurement of ABTS ^+^ · clearance

Following the method of Johnston et al.[22] with slight modifications. Prepare potassium persulfate into a stock solution of 2.6 mmol/L, add 7.4 mmol/L ABTS solution, mix well, stand in the dark for 16 h, measure the absorbance at 734 nm to be A1, add absolute ethanol instead of ABTS ^+^ solution to sample to measure the absorbance to be A2, and add pure water instead of sample to ABTS ^+^ solution to be A0.

#### 2.10.3 Measurement of antioxidant activity by iron reduction/antioxidant capacity (FRAP)

The FRAP working solution (consisting of 300 mmol/L acetate buffer pH 3.6, 10 mmol/L TPTZ, and 20 mmol/L FeCl_3_ in a ratio of 10:1:1) was mixed with the sample solution, and after avoiding light and reacting, the absorbance was measured at 593 nm. Plot a standard curve using Trolox as the standard, and express the results in terms of Trolox equivalents.

### 2.11 Determination of antioxidant activity of cells

#### 2.11.1 Cell grouping and methods of administration

The HepG2 cells in the logarithmic growth phase were selected. The cells were divided into blank group, T-BHP group, VC control group (20 µg·mL^−1^), low dose (50 µg·mL^−1^) of FDF, medium dose (100 µg·mL^−1^) of FDF, and high dose (200 µg·mL^−1^) of FDF. The FDF group was treated with corresponding quantities of FDF for 24 h, and the blank group and T-BHP group were replaced with serum-free MEM medium. After the treatment, 400 µM T-BHP was added, and then the culture medium was discarded for treatment for 2 h.

#### 2.11.2 Determination of intracellular antioxidant enzyme activity

Cells were seeded and cultured according to the protocol described in Section 2.11.1, with each experimental group performed in triplicate. Cell lysates were prepared using Western and IP Lysis Buffer (as per manufacturer’s instructions), and supernatants were collected after centrifugation (12,000 × g, 15 min, 4 °C). Total protein concentration was determined via the BCA assay. Intracellular MDA, CAT, SOD, and GSH-Px activities or concentrations were quantified using commercial assay kits following the manufacturers’ protocols; all values were normalized to total protein content in the corresponding lysate.

#### 2.11.3 Western blot detection

Total proteins were extracted from cells or tissues using RIPA lysis buffer supplemented with protease and phosphatase inhibitors. Protein concentration was quantified via the BCA assay. Equal amounts of protein (20–40 μg per lane) were resolved by SDS-PAGE and electrophoretically transferred onto PVDF membranes. Membranes were blocked for 1 h at room temperature with 5 % (w/v) non-fat dry milk in TBST, then incubated overnight at 4 °C with primary antibodies against Nrf2, HO-1, and NQO1 (all diluted 1:1000 in blocking buffer). After three 10-min washes with TBST, membranes were incubated for 1 h at room temperature with HRP-conjugated secondary antibodies (diluted 1:5000). Immunoreactive bands were visualized using ECL chemiluminescence reagent, and β-actin or GAPDH served as loading controls. Band intensities were quantified using ImageJ software (NIH), and target protein expression was normalized to the corresponding loading control.

### 2.12 Data processing

Statistical significance was assessed using one-way ANOVA with Tukey’s post hoc test (*p* < 0.05) in GraphPad Prism 10.3.0. Response surface methodology (RSM) modeling and optimization were conducted using Design-Expert 13. All experiments were performed in triplicate, and reported values represent the mean ± standard deviation of three independent replicates.

## 3 Results and discussion

### 3.1 Analysis of non-targeted metabolomics of four species of *Dendrobium*

**Fig. 1:**
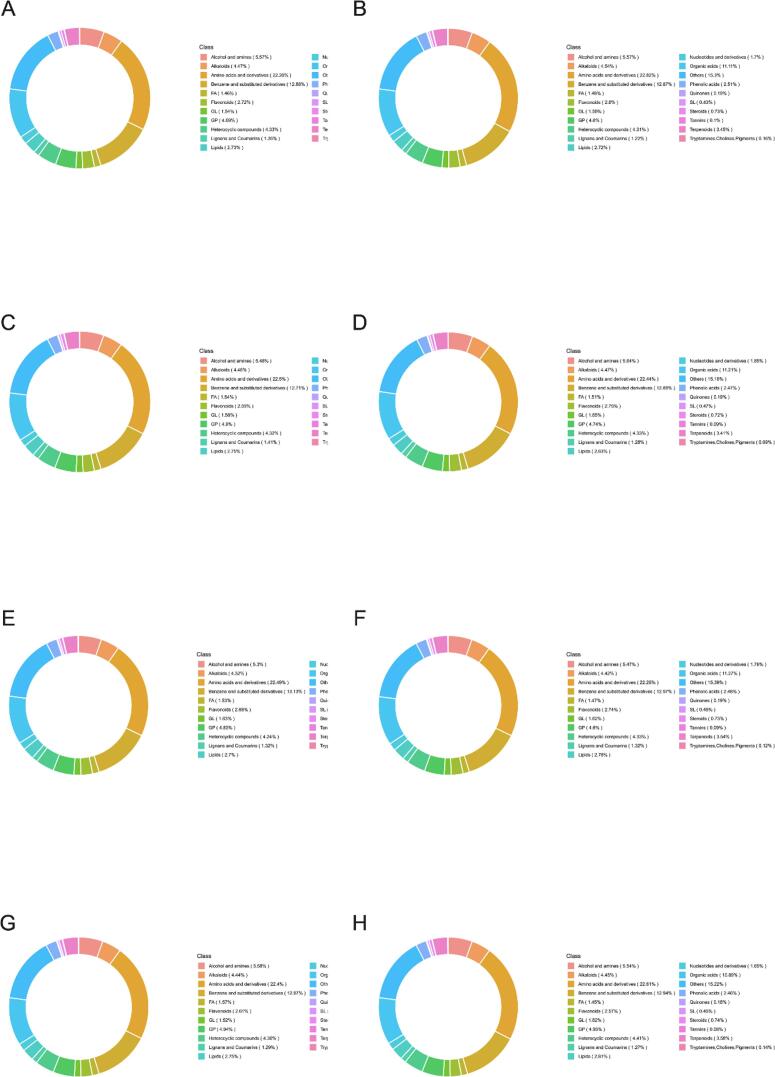
The metabolite profiles of the four Dendrobium species under alcohol and water extraction were analyzed by untargeted metabolomics. The complete metabolite composition data for each sample are provided in Supplementary Figs. S1-S8(eight individual figures for each extraction condition and species).

The metabolite profiles of Dendrobium flexicaule, Dendrobium huoshanense, and 3- versus 5-year-old Dendrobium officinale were systematically compared under parallel aqueous and ethanolic extraction conditions to identify both the optimal plant material (i.e., species and age) and the most efficient extraction solvent for maximizing flavonoid solubilization. Untargeted metabolomic analysis revealed that the global metabolic architecture across all samples was highly conserved, with amino acids and derivatives, benzenoids, and organic acids consistently representing the three most abundant chemical classes. Nevertheless, flavonoid abundance exhibited significant variation driven jointly by botanical origin (species and ontogenetic stage) and extraction modality (aqueous vs. ethanolic) thereby reflecting intrinsic biochemical specificity and differential solvent–metabolite interaction efficiency (Fig. 1).

Under the condition of alcohol extraction, the content of flavonoids in *Dendrobium flexicaule* was 2.72 %, which was higher than that in *Dendrobium huoshan*.

Under ethanolic extraction, flavonoid contents were 2.59 % in *Dendrobium flexicaule*, 2.68 % in 3-year-old *Dendrobium officinale*, and 2.61 % in 5-year-old *Dendrobium officinale* indicating that ethanolic extraction achieved maximal flavonoid solubilization across all tested materials. In contrast, under aqueous extraction, flavonoid content in D. flexicaule was 2.59 %, significantly lower than in 3-year-old D. officinale (2.74 %) and D. huoshanense (2.75 %), but marginally higher than in 5-year-old D. officinale (2.57 %). Collectively, these data demonstrate that D. flexicaule exhibits superior flavonoid extractability specifically under ethanolic conditions suggesting a higher proportion of ethanol-soluble aglycones or low-polarity glycosides and thus represents a preferential source for organic solvent–based flavonoid isolation.

The influence of extraction solvent on flavonoid solubilization exhibited species- and age-dependent divergence: ethanolic extraction yielded significantly higher flavonoid recovery than aqueous extraction for *Dendrobium flexicaule* and 5-year-old *Dendrobium officinale*, whereas aqueous extraction outperformed ethanolic extraction for *Dendrobium huoshanense* and 3-year-old D. officinale. This dichotomous behavior is mechanistically attributable to interplay among three factors flavonoid aglycone/glycoside composition (governing intrinsic polarity), cell wall polysaccharide architecture (affecting solvent penetration and mass transfer), and ontogenetic stage (modulating both metabolite profile and tissue lignification) [23], [24], [25], [26]. It was found that ethanol concentration had a significant effect on the dissolution efficiency of flavonoid glycosides, and an appropriate proportion of organic solvents was more conducive to flavonoid release [24]. The distribution of flavonoids in different tissues and the differences in extraction efficiency are closely related to their polarity [25]. In addition, growth years regulate the accumulation of secondary metabolites, and there are differences in the expression of genes related to flavonoid synthesis in *Dendrobium huoshanense* in different years, explaining the differences in flavonoid content and optimal technology between 3 and 5 years [26]. The composition pattern of flavonoids is also influenced by genetic background and environmental factors [23].

Comprehensive comparative analysis confirmed that *Dendrobium flexicaule* exhibited the highest flavonoid solubilization yield under ethanolic extraction conditions, establishing it as a superior botanical source for flavonoid enrichment. Subsequent work will therefore prioritize process optimization of ethanolic extraction specifically for D. flexicaule. Key parameters including ethanol concentration, ultrasonic temperature duration, and solid-to-liquid ratio will be systematically evaluated via single-factor experiments followed by response surface methodology (RSM) using a central composite design. The resulting optimized protocol will deliver both high flavonoid recovery and inter-batch reproducibility, thereby providing a robust technical foundation for scalable flavonoid isolation, functional ingredient development, and evidence-based refinement of D. flexicaule processing standards and quality specifications.

### 3.2 Optimization of extraction conditions

#### 3.2.1 Single factor experimental results

**Fig. 2:**
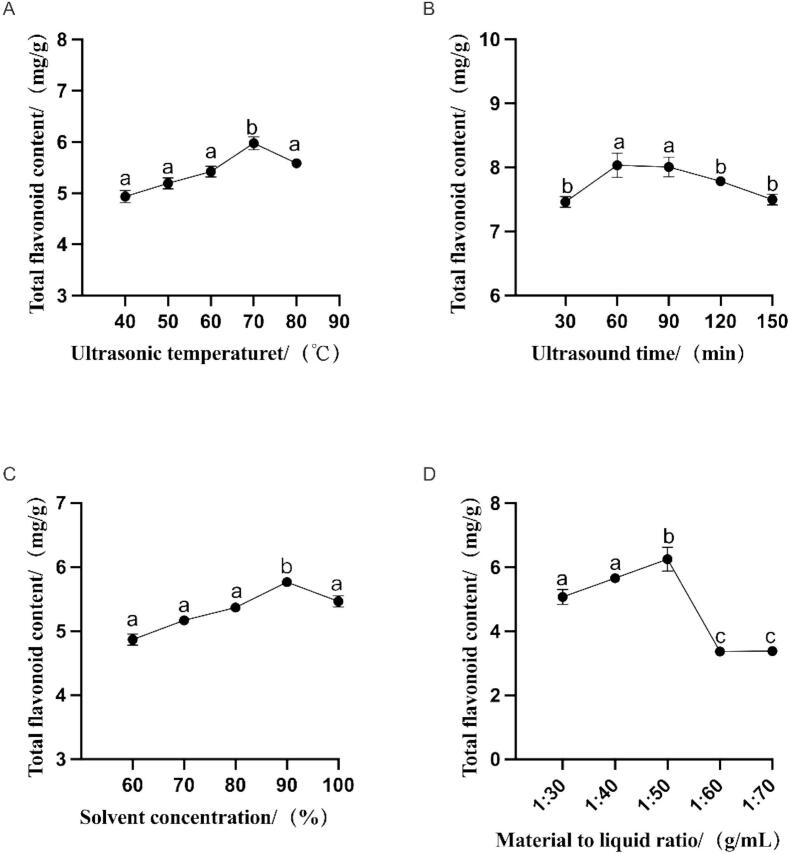
Single-factor experimental plot.

As shown in Fig. 2A–2D, the flavonoid dissolution yield from *Dendrobium flexicaule* (FDF) increased progressively with rising ultrasonic temperature from 40 °C to 70 °C, peaked at 70 °C, and then declined markedly at 80 °C indicating thermal degradation of thermolabile flavonoid glycosides [27]. The flavonoid dissolution yield increased markedly between 30 and 60 min, peaked at 60 min, and then declined progressively thereafter.. This decline is attributable to ultrasonication-induced structural degradation of thermolabile flavonoid glycosides and/or co-extraction of non-flavonoid matrix components both factors that compromise assay specificity and quantitative accuracy [28]. As the ethanol volume fraction increased from 60 % to 90 %, the flavonoid dissolution yield increased progressively and peaked at 90 % ethanol. Further increasing the ethanol concentration to 100 % led to a statistically significant decline in yield attributable to competitive solubilization of highly polar, non-flavonoid matrix constituents (e.g., organic acids and glycosylated phenolics), which compromises assay specificity and reduces effective flavonoid recovery [29]. The flavonoid dissolution yield increased progressively with increasing material-to-liquid ratio from 30:1 to 50:1 and peaked at 50:1. Beyond this optimum, further elevation of the ratio led to a statistically significant decrease in yield attributable to disproportionate co-extraction of non-flavonoid matrix constituents, particularly polysaccharides and hydrolysable tannins, which interfere with flavonoid quantification by reducing chromatographic peak resolution and inducing matrix-related signal suppression [30]. Therefore, the optimal extraction conditions for flavonoid recovery from *Dendrobium flexicaule* are 70 °C, 60  min, 90 % ethanol (v/v), and a material-to-liquid ratio of 50:1 (g/mL).

#### 3.2.2 Model fitting and response surface analysis

Preliminary single-factor experiments evaluated the effects of four key variables ultrasonic temperature (A), ultrasonic time (B), ethanol concentration (C), and material-to-liquid ratio (D) on the total flavonoid yield from *Dendrobium flexicaule*. Subsequently, a four-factor, three-level Box–Behnken design (BBD) was employed for response surface methodology (RSM) optimization (Table 3). A quadratic regression model was established to predict flavonoid yield, and statistical significance of model terms was assessed via analysis of variance (ANOVA).

**Table 3:** Response surface experimental design and results.

Test No.	A- ultrasonic temperature (℃)	B-Extraction time (min)	C-Ethanol Volume Fraction (%)	D-Feed to Liquid Ratio (g/mL)	Flavonoids extracted (mg/g)
1	60	30	90	50	5.82
2	80	30	90	50	7.53
3	60	90	90	50	6.48
4	80	90	90	50	3.72
5	70	60	80	40	4.55
6	70	60	100	40	5.53
7	70	60	80	60	4.52
8	70	60	100	60	4.28
9	60	60	90	40	7.73
10	80	60	90	40	4.14
11	60	60	90	60	3.47
12	80	60	90	60	5.88
13	70	30	80	50	6.01
14	70	90	80	50	4.82
15	70	30	100	50	6.17
16	70	90	100	50	6.42
17	60	60	80	50	6.73
18	80	60	80	50	3.59
19	60	60	100	50	4.91
20	80	60	100	50	6.93
21	70	30	90	40	4.52
22	70	90	90	40	5.83
23	70	30	90	60	5.26
24	70	90	90	60	4.50

25	70	60	90	50	8.51
26	70	60	90	50	8.48
27	70	60	90	50	8.53
28	70	60	90	50	8.02
29	70	60	90	50	8.81

The regression equations obtained were as follows: Y = 8.44 – 0.2667a-0.2917b + 0.3250c-0.3667d-0.10ab + 1.27AC + 1.53AD + 0.3750bc-0.5000bd-0.3250 cd-1.25a^2^-1.24B^2^-1.57C^2^-2.10D^2^.

As shown in Table 4, the regression model exhibits a high F-value of 22.01 and a statistically significant p-value (p < 0.01), confirming its overall significance at the *p* < 0.01 level. The lack-of-fit term was non-significant (*p* = 0.0996 > 0.05), indicating that the quadratic model fits the experimental data well and that random experimental error is minimal. The coefficient of variation (CV) was 8.13 % (<10 %), indicating minimal influence of non-experimental variability and high model stability. The determination coefficient (R2) was 0.9565, signifying that the quadratic model accounts for 95.65 % of the total variation in the experimental flavonoid yield demonstrating an excellent fit to the observed data and robust representation of factor–response relationships. The adjusted coefficient of determination was 0.9131, which is close to the unadjusted R2 (0.9565), further supporting the model’s reliability and its suitability for predicting the effects of individual factors on flavonoid yield.

**Table 4:** Analysis of variance for regression simulation.

Source	Sum of squares of dispersions	Degree of freedom	mean square	F value	P-value	Significance
Models	0.7	14	0.05	22.01	< 0.0001	**
A- ultrasonic Temperature	0.008533	1	0.008533	3.72	0.0741	
B-Extraction time	0.0102	1	0.0102	4.46	0.0533	
C-Ethanol volume fraction	0.0127	1	0.0127	5.53	0.03381	*
D-Feed-Liquid Ratio	0.0161	1	0.0161	7.04	0.0189	*
AB	0.0484	1	0.0484	21.12	0.0004	**
AC	0.065	1	0.065	28.38	0.0001	**
AD	0.093	1	0.093	40.6	< 0.0001	**
BC	0.005625	1	0.005625	2.45	0.1395	
BD	0.001	1	0.001	4.36	0.0554	
CD	0.004255	1	0.004255	1.84	0.1960	
A^2^	0.10	1	0.10	44.47	< 0.0001	**
B^2^	0.10	1	0.10	43.59	< 0.0001	**
C^2^	0.16	1	0.16	69.41	< 0.0001	**
D^2^	0.29	1	0.29	125.24	< 0.0001	**
residual	0.03	14	0.002291			
Misfit term	0.03	10	0.002876	3.46	0.1212	ns
Pure error	0.00332	4	0.00083			
total	0.73	28				
R^2^ = 0.9565 Adj R^2^ = 0.9131 Pred R^2^ = 0.7685						

Note: P < 0.01 is extremely significant, indicated by **; P < 0.05 is significant, indicated by *; P > 0.05 is not significant, indicated by ns.

As shown in Table 3, the interaction term A × D and all quadratic terms (A^2^, B^2^, C^2^, D^2^) exerted highly significant effects on flavonoid yield (*p* < 0.01). The interaction terms A × B and A × C also showed statistically significant effects (*p* < 0.05), whereas all other linear and interaction terms were non-significant (*p* > 0.05). Furthermore, based on standardized regression coefficients and *p*-values, the relative influence of the four factors on total flavonoid yield from *Dendrobium flexicaule* follows the order: material-to-liquid ratio (D) > ethanol concentration (C) > ultrasonic time (A) > ultrasonic temperature (B).

**Fig. 3:**
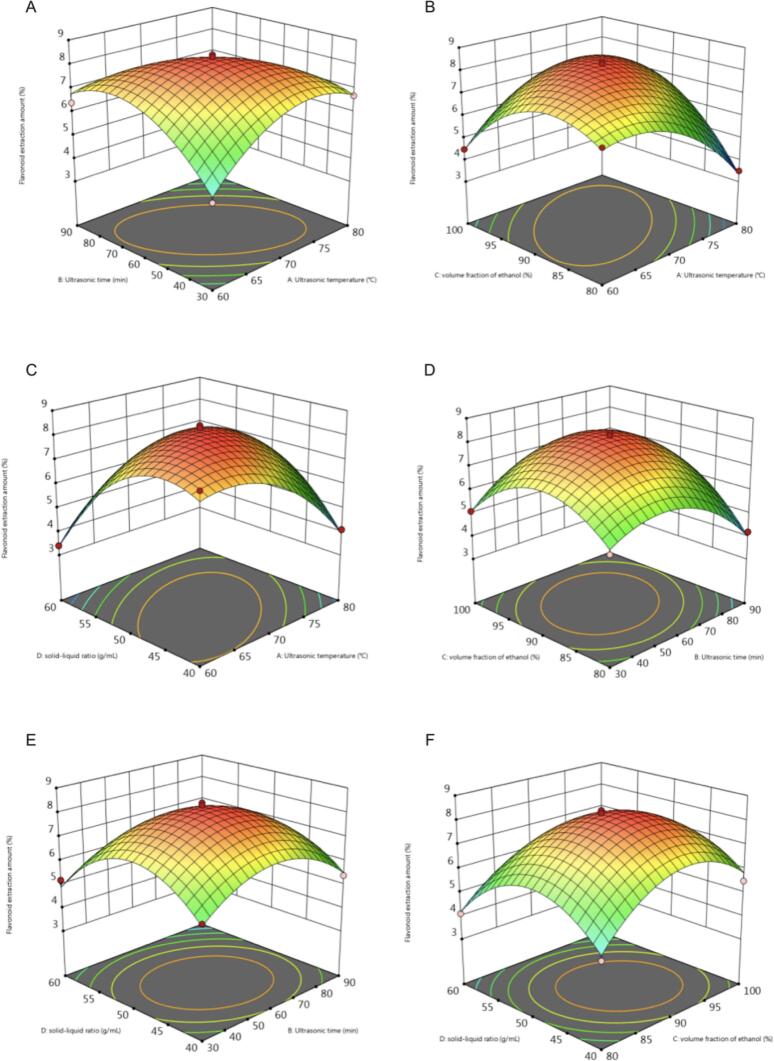
Response surface plot of total flavonoids extracted from *Dendrobium flexicaule* under the interaction of various factors.

Response surface analysis revealed that ultrasonic temperature, ultrasonic time, ethanol concentration, and material-to-liquid ratio all exerted statistically significant effects on flavonoid yield (*p* < 0.01). Each factor exhibited a quadratic (parabolic) relationship with the response, and all two-factor interactions were significant at the *p* < 0.01 level. The elliptical contours observed in Fig. 3 further confirmed the interdependence of factor effects on flavonoid yield, with greater ellipticity indicating stronger interaction intensity between the corresponding factors.

Among the interaction terms, A × D (ultrasonic temperature × material-to-liquid ratio) exhibited the strongest effect (p < 0.0001; Table 4). This pronounced interaction arises from the synergistic interplay between temperature-dependent mass transfer kinetics and solvent volume–mediated extraction driving force. Elevated ultrasonic temperature increases the diffusion coefficient of flavonoids, thereby accelerating their release from the plant matrix. Critically, this thermal enhancement is amplified under higher material-to-liquid ratios: the larger solvent volume sustains a steeper concentration gradient between the solid phase and bulk solvent, further promoting mass transfer. In contrast, at lower material-to-liquid ratios, the extractant phase approaches saturation more rapidly, attenuating the beneficial effect of increased temperature on flavonoid solubilization and diffusion.

The A × B interaction (ultrasonic temperature × ultrasonic time) was also statistically significant (*p* < 0.001). This interaction reflects the competing effects of ultrasound-induced cavitation and thermal degradation on flavonoid stability. Prolonged ultrasonic treatment initially enhances cell wall disruption and flavonoid release via cavitation-generated microjets and shear forces. However, extended exposure to elevated temperatures particularly above 70 °C promotes thermal degradation of heat-labile flavonoids, as confirmed by the reduced yield observed at 80 °C in the single-factor experiment (Fig. 2A). Consequently, the combined effect of temperature and time follows an optimum (quadratic) response rather than a monotonic trend.

The A × C interaction (ultrasonic temperature × ethanol concentration) was statistically significant (*p* < 0.001). This interaction arises from temperature-dependent modulation of solvent polarity in ethanol–water mixtures. The dielectric constant and thus the overall polarity of these binary solvents decrease with increasing temperature, thereby improving the solubility of moderately non-polar flavonoid aglycones. However, at high ethanol concentrations (≥90 % v/v), the solvent system becomes excessively non-polar, reducing its capacity to solubilize hydrophilic flavonoid glycosides and potentially inducing their precipitation. Consequently, the optimal ultrasonic temperature for maximal flavonoid yield is not fixed but varies systematically with ethanol concentration a hallmark of a significant interaction effect.

Notably, the predicted R^2^ (0.7685) was substantially lower than the adjusted R^2^ (0.9131), with a difference of 0.145. This discrepancy indicates reduced predictive capacity of the quadratic model for new observations and suggests potential overfitting i.e., the model captures noise or dataset-specific artifacts rather than the underlying factor–response relationship. Given this limitation of the conventional RSM framework, we subsequently evaluated machine learning alternatives; among these, the support vector regression (SVR) model demonstrated markedly superior performance, achieving an R^2^ of 0.9893 and significantly enhanced predictive robustness (Section 3.2.3).

Given the significant synergistic and antagonistic interactions among extraction factors as evidenced by the statistically significant two-factor interaction terms (A × D, A × B, A × C) and quadratic effects in Table 4 the single-factor-at-a-time (SFAT) approach is inadequate for identifying the true global optimum. Consequently, response surface methodology (RSM) was employed to optimize the multivariable extraction system. Under the quadratic RSM model, the predicted optimal conditions for maximal flavonoid yield were: ultrasonic temperature = 68 °C, ultrasonic time = 60  min, ethanol concentration = 90 % (v/v), and material-to-liquid ratio = 1:48  g/mL.

Notably, the predicted R^2^ (0.7685) was substantially lower than the adjusted R^2^ (0.9131), with an absolute difference of 0.145. This discrepancy indicates diminished predictive capacity for external validation data and suggests potential overfitting i.e., the quadratic model captures dataset-specific noise rather than the underlying factor–response relationship. Although the high adjusted R^2^ confirms a strong fit to the calibration dataset, the markedly lower predicted R^2^ reveals limited generalizability to new experimental conditions. Given this inherent constraint of conventional response surface methodology (RSM), we pursued machine learning–based alternatives. As detailed in Section 3.2.3, the support vector regression (SVR) model achieved an R^2^ of 0.9893 and exhibited minimal divergence between training and cross-validated performance, confirming its superior predictive robustness for modeling flavonoid yield under varying extraction conditions.

#### 3.2.3 Evaluation of prediction performance of machine learning model

##### 3.2.3.1 Correlation analysis of variables

**Fig. 4:**
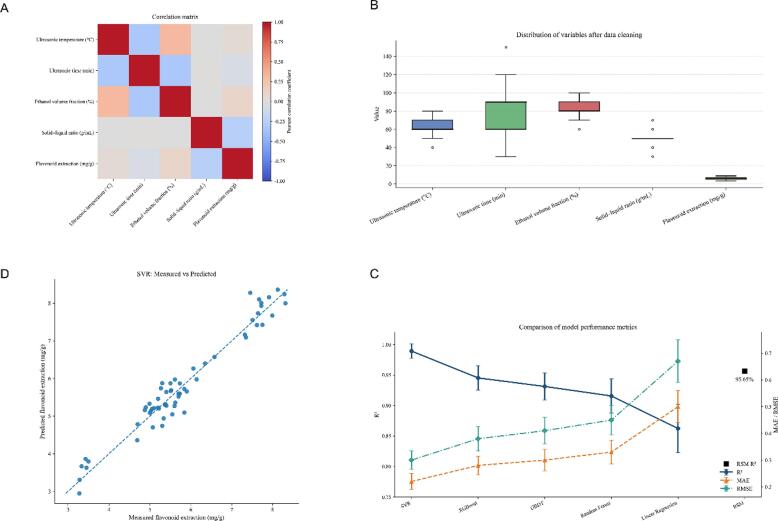
Data analysis and model evaluation of flavonoid extraction prediction.

Pearson correlation coefficients were calculated to quantify linear associations among the four process variables ultrasonic temperature (A), ultrasonic time (B), ethanol volume fraction (C), material-to-liquid ratio (D) and flavonoid yield. The resulting correlation heatmap is presented in Fig. 4A. All pairwise correlations among input variables had absolute values < 0.30, confirming their low mutual collinearity and validating the experimental design’s orthogonality. This minimizes multicollinearity-induced instability in model coefficient estimation. In contrast, the correlations between individual factors and flavonoid yield ranged from 0.22 to 0.39, indicating weak-to-moderate linear relationships. Critically, the absence of strong univariate correlations coupled with statistically significant interaction and quadratic terms in the RSM model (Table 4) demonstrates that flavonoid extraction is governed by complex, nonlinear factor interdependencies rather than simple additive effects. This mechanistic insight provides a rigorous justification for adopting nonlinear modeling approaches, including machine learning algorithms, to capture the true underlying response surface.

##### 3.2.3.2 Data distribution characteristics

Box plots were generated for ultrasonic temperature (A), ultrasonic time (B), ethanol volume fraction (C), material-to-liquid ratio (D), and flavonoid yield to assess data distribution, dispersion, and the presence of potential outliers following data cleaning. As shown in Fig. 4B, all variables exhibit reasonable range coverage with no extreme outliers defined as points beyond 1.5 × IQR from the nearest quartile confirming overall experimental data stability. A small number of mild outliers (i.e., points outside the whiskers but within 3 × IQR) were observed for several variables; however, these fall within statistically acceptable limits and show no evidence of measurement error or systematic bias. Furthermore, the interquartile ranges (IQRs) and median positions vary substantially across variables, reflecting deliberate variation in the experimental design. This intentional amplitude in factor and response levels ensures sufficient information content for robust model training and facilitates reliable identification of underlying relationships between process parameters and flavonoid yield.

##### 3.2.3.3 Model performance comparison

The predictive performance of linear regression (LR), support vector regression (SVR), random forest regression (RF), gradient boosting decision tree (GBDT), and XGBoost regression was systematically evaluated against the conventional quadratic response surface methodology (RSM) using a rigorous nested cross-validation framework with grouped splitting designed to prevent data leakage and ensure unbiased generalization assessment. As shown in Fig. 4C, substantial differences in both prediction accuracy and stability were observed across models. All machine learning based nonlinear models outperformed LR and RSM across key metrics: R^2^ (coefficient of determination), MAE (mean absolute error), and RMSE (root mean square error). This consistent superiority confirms that the relationship between extraction parameters and flavonoid yield is inherently nonlinear and cannot be adequately captured by linear or low-order polynomial approximations. Model performance metrics are summarized in Table 5.

**Table 5:** Comparison of prediction performance of different models.

Models	R2 (%)	MAE (mg/g)	RMSE (mg/g)
RSM	95.65	0.124	0.168
LR	82.37	0.231	0.297
SVR	98.93	0.058	0.082
RF	94.26	0.136	0.179
GBDT	95.18	0.121	0.162
XGBoost	96.42	0.103	0.141

Among all evaluated models, the support vector regression (SVR) model achieved the highest predictive performance, with an R^2^ of 0.9893, the lowest MAE (0.058 mg/g), and the lowest RMSE (0.082 mg/g) demonstrating superior accuracy in both model fitting and external prediction. Notably, the SVR R^2^ exceeds that of the quadratic RSM model by 0.2762 (i.e., 27.62 percentage points), confirming its substantially enhanced capacity to capture the complex, nonlinear mapping between extraction parameters and flavonoid yield. This marked improvement underscores the advantage of SVR in modeling systems governed by multifactorial interactions and non-additive effects, as evidenced by the significant A × B, A × C, and A × D interaction terms in the RSM analysis (Table 4).

Decision tree-based ensemble learning models such as XGBoost, GBDT, and RF also show good prediction ability. However, these models recorded a high level overall, but still slightly lower than the SVR model with R^2^ values are 96.42 %, 95.18 %, and 94.26 %, respectively. This may be related to the relatively limited sample size and fewer variable dimensions, in which case the generalization ability of SVR for small sample nonlinear problems is more advantageous. In contrast, the linear regression model has the lowest R^2^ (82.37 %) and higher MAE (0.231 mg/g) and RMSE (0.297 mg/g), which indicates that it is difficult to effectively describe the interaction and nonlinear effects between process factors.

Collectively, the machine learning models particularly SVR demonstrate superior predictive capability compared to both linear regression and conventional quadratic response surface methodology (RSM), as consistently evidenced by higher R2, lower MAE, and lower RMSE across rigorous nested cross validation (Fig. 4C; Table 5). Among the evaluated ML algorithms, SVR achieved the highest R2 (0.9893) and lowest error metrics, establishing it as the most robust predictor of flavonoid yield under varying extraction conditions. This performance advantage, grounded in SVR’s capacity to model complex, nonlinear input–output relationships with limited data, underscores its strong potential for accurate process modeling and parameter optimization in natural product extraction.

##### 3.2.3.4 Model prediction consistency analysis

Having identified SVR as the optimal predictive model, we further assessed its agreement with experimental observations by plotting predicted versus measured flavonoid yield values (Fig. 4D). The scatterplot shows tight clustering of data points around the parity line (y = x), with a high coefficient of determination (R^2^ = 0.9893) and a narrow residual distribution confirming excellent quantitative agreement between predictions and measurements. Only three outliers (residuals > 0.12 mg/g) were observed, all within ± 0.15 mg/g, and no systematic bias (e.g., heteroscedasticity or trend in residuals) was detected. This demonstrates SVR’s strong capacity to characterize not only the magnitude but also the directional trend of flavonoid yield variation across the full range of experimental process conditions.

The linear regression of predicted versus measured flavonoid yield values yielded an equation of y = 0.987x + 0.124 (R2 = 0.989, RMSE = 0.082 mg/g, MAE = 0.058 mg/g), confirming the SVR model’s high predictive accuracy and minimal systematic bias evidenced by the near-unity slope (0.987) and small intercept (0.124 mg/g). These results further validate the SVR model’s stability and generalization capability across the full dataset demonstrating not only superior aggregate performance (as quantified by R^2^, MAE, and RMSE in Table 5) but also consistently reliable point wise predictions for individual samples (Fig. 4D). This dual-level robustness aligns precisely with the comparative model evaluation outcomes presented earlier, reinforcing the conclusion that the SVR model, as a machine learning based nonlinear regressor, offers distinct advantages in capturing the complex, multifactorial relationship between extraction parameters and flavonoid yield. The SVR-optimized extraction conditions were predicted as follows: ultrasonic temperature = 71.65 °C, ultrasonic time = 67.66 min, ethanol volume fraction = 94.03 % (v/v), and material-to-liquid ratio = 1:49.86 (w/v). Under these conditions, the model predicts a maximum flavonoid yield of 8.93 mg/g.

#### 3.2.4 Validation of RSM and SVR models

The SVR model achieves a higher R^2^ (0.9893) than the quadratic RSM model (R^2^ = 0.7131), confirming its superior predictive accuracy and fidelity to experimental observations. To ensure operational feasibility, the SVR-predicted optimal conditions were marginally adjusted specifically, ultrasonic temperature from 71.65 °C to 72 °C, ultrasonic time from 67.66 min to 68 min, ethanol volume fraction from 94.03 % (v/v) to 94 % (v/v), and material-to-liquid ratio from 1:49.86 (w/v) to 1:50 (w/v) all within ± 0.5 % of the original optimized values. Under these practically implemented conditions, the experimentally measured flavonoid yield was 8.90 mg/g, closely matching the SVR prediction of 8.93 mg/g (absolute error = 0.03 mg/g; relative error = 0.34 %). In contrast, the RSM-predicted optimum (ultrasonic temperature = 68 °C, time = 60 min, ethanol = 90 % (v/v), ratio = 1:48 (w/v)) yielded an experimental value of 8.26 mg/g 3.9 % lower than its own prediction of 8.49 mg/g. This consistent underprediction by RSM, coupled with its substantially lower R2 and higher residual error (RMSE = 0.297 mg/g vs. SVR’s 0.082 mg/g), demonstrates that SVR provides not only improved point prediction but also greater reliability across the entire experimental domain.

### 3.3 FDF purification

The purity of the flavonoid-enriched fraction (FDF) increased significantly from 12.4 % (w/w) prior to purification to 25.8 % (w/w) after AB-8 macroporous resin chromatography yielding a purification fold of 2.08. This twofold enrichment confirms the efficacy of the AB-8 resin-based protocol in selectively concentrating flavonoids while removing co-extracted impurities, thereby substantially improving product quality and rendering the FDF suitable for downstream antioxidant activity assays.

### 3.4 Identification of FDF components

A total of 34 flavonoids were identified in the flavonoid enriched fraction (FDF), providing a chemically grounded basis for its experimentally observed antioxidant activity (Table 2). Among these, flavonols were the most abundant subclass comprising 10 compounds, with rutin as the dominant constituent (29.99 mg/g FDF) and collectively represented 33.49 % of the total quantified flavonoid content. There were 10 kinds of flavonoids (the highest content of narcisin, 3.23), accounting for 5.77 % of the total flavonoids. There are 4 kinds of chalcones (among them, naringenin contains the highest chalcone content, 8.29), accounting for 21.21 % of the total flavonoids. There were three kinds of dihydroflavonoids (the highest content of sinfol, 20.66), accounting for 21.16 % of the total flavonoids. There are two kinds of dihydroflavonols (the highest content of doughnut is 3.61), accounting for 4.00 % of the total flavonoids. There are three kinds of isoflavones (among which O-methylated isoflavones have the highest content, 0.09), accounting for 0.15 % of the total flavones. There is one kind of flavonoid carboglycosides (vitexin, 0.02), accounting for 0.22 % of the total flavonoids. The FDF are mainly flavonols, chalcones, and dihydroflavonoids, which together account for more than 75 % from the perspective of composition and structure. [31]reported that flavonols (such as quercetin, kaempferol derivatives) have strong free radical scavenging ability and are the main contributors to the antioxidant activity of plant extracts. Chalcones also exhibit significant antioxidant and anti-inflammatory activities due to their unique α, β-unsaturated ketone structures [32]. Dihydroflavonoids also have certain advantages in scavenging free radicals due to the saturated structure of the C-ring[33]. The higher proportion of chalcones and dihydroflavonoids in *Dendrobium flexicaule* may be related to its unique genetic background and secondary metabolic regulation compared with other *Dendrobium* plants [34]. The diversity and high content of the above-mentioned flavonoids provide an important chemical basis for the further development of the alcohol extract of *Dendrobium flexicaule* as a natural antioxidant or functional food ingredient. Subsequently, the activity of the main flavonoid monomers can be tracked and separated, while the antioxidant efficacy evaluation at the cellular and animal levels can be carried out to clarify the pharmacodynamic substance basis and mechanism of action.

### 3.5 Antioxidant capacity of FDF in vitro

**Fig. 5:**
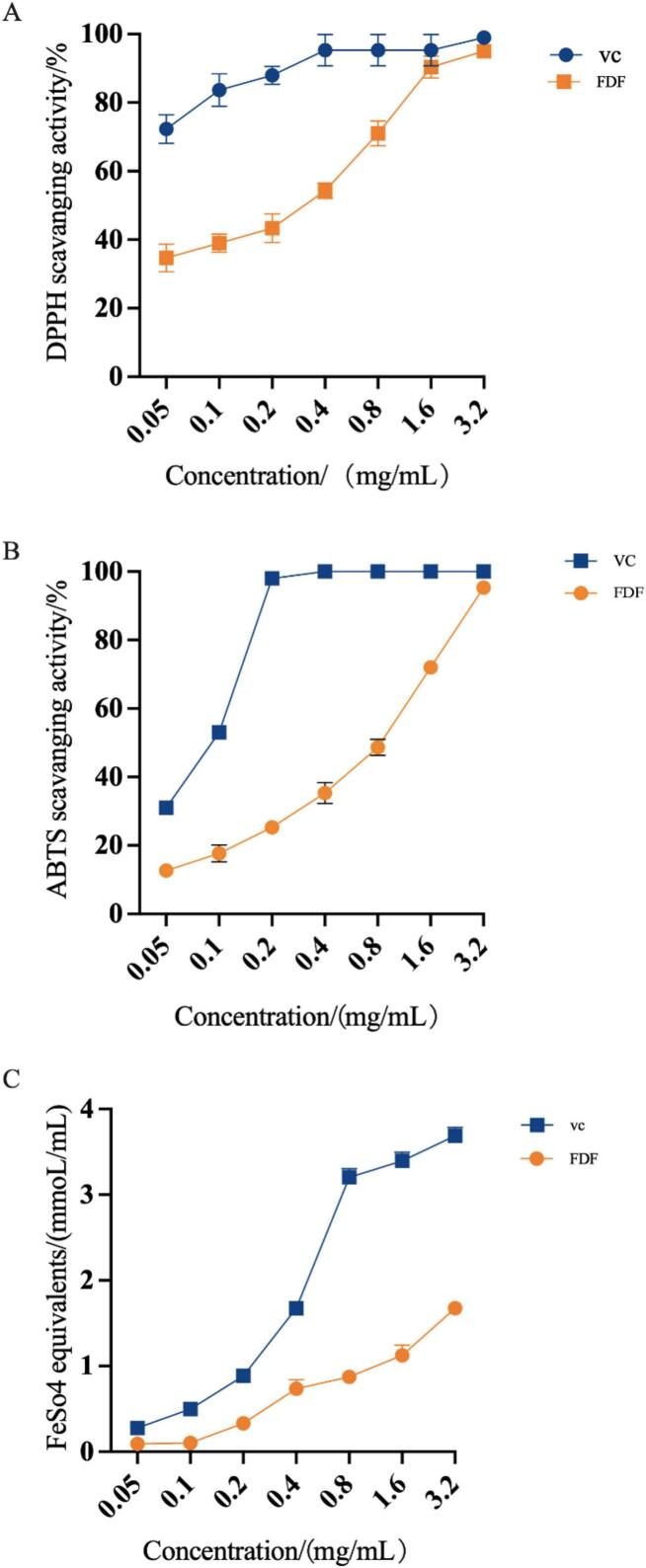
In vitro antioxidant capacity.

The in vitro antioxidant activity of FDF was comprehensively evaluated using the DPPH radical scavenging assay. Results from the ABTS^+^ radical scavenging and ferric ion (Fe^3+^) reducing power assays are presented in Fig. 5. FDF exhibited dose-dependent scavenging activity against both DPPH and ABTS^+^ radicals. At the highest tested concentration, FDF achieved scavenging rates comparable to those of the positive control vitamin C (VC), though its ABTS^+^ scavenging efficacy remained slightly lower than that of VC. Similarly, the Fe^3+^ reducing capacity of both FDF and VC increased in a concentration-dependent manner. However, at equivalent concentrations, VC exhibited a significantly higher ferrous ion (Fe^2+^) equivalent value than FDF indicating superior reducing power. Collectively, these data demonstrate that FDF possesses potent free radical scavenging activity but comparatively modest reducing capacity.

### 3.6 The protective effect of FDF on oxidative damage induced by t-BHP in HepG2 cells

**Fig. 6:**
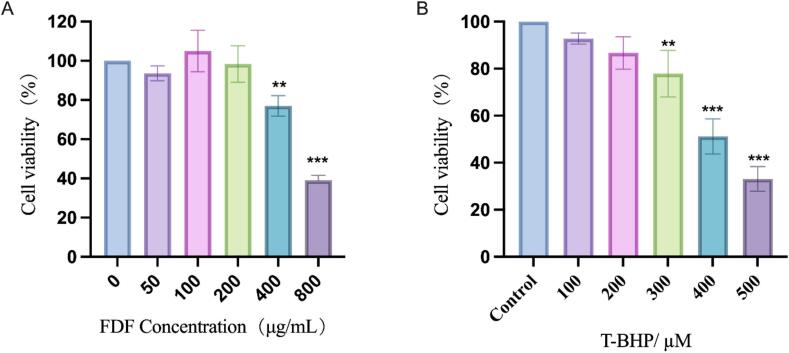
Oxidative Stress Model of hepg2 Cells and Effects of Different Concentrations of FDF on Cell Viability.

**Fig. 7:**
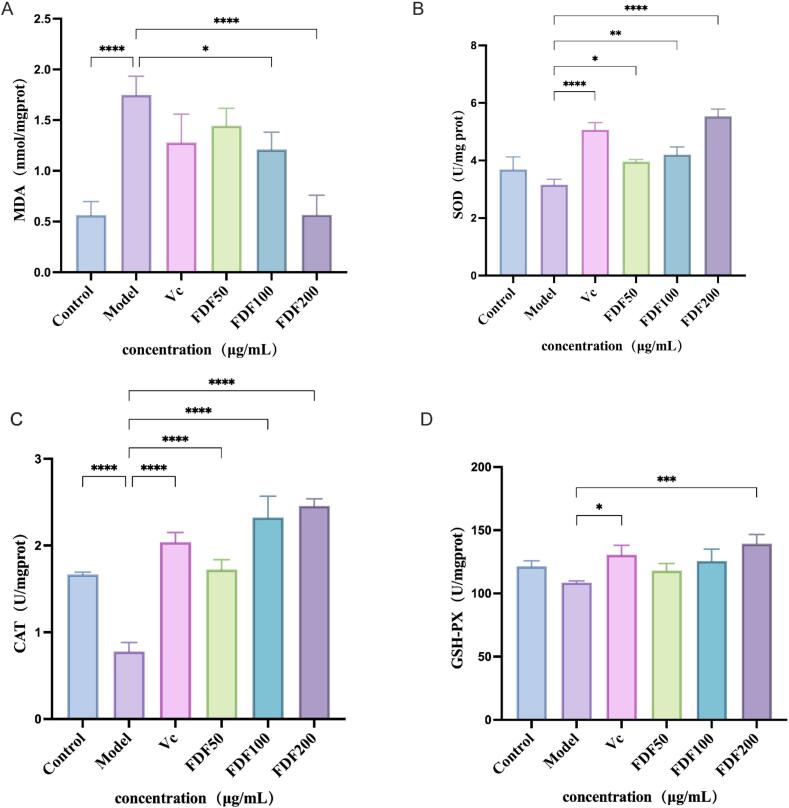
Effects of different concentrations of FDF on oxidative stress in hepg2 cells.

The cellular oxidative stress model was established according to the method described by Wu et al. [21], as shown in Fig. 6. Intracellular MDA levels widely accepted as a biomarker of lipid peroxidation serve as an indirect indicator of oxidative damage in HepG2 cells [35]. Fig. 7A shows that intracellular MDA levels in the t-BHP–treated model group were significantly higher than those in the untreated blank group after 2 h of stimulation. Both FDF and vitamin C (VC) treatment significantly suppressed oxidative stress–induced MDA accumulation. The difference was highly significant relative to the model group (*P* < 0.01), and inhibition was concentration-dependent. Superoxide dismutase (SOD), glutathione peroxidase (GSH-Px), and catalase (CAT) constitute the core enzymatic antioxidant defense system in HepG2 cells; changes in their activity are closely associated with both oxidative stress–induced damage and antioxidant protection mechanisms. SOD catalyzes the disproportionation of superoxide radicals into molecular oxygen and hydrogen peroxide (H_2_O_2_), serving as a primary enzymatic defense against superoxide anions. CAT subsequently decomposes H_2_O_2_ into water and molecular oxygen, whereas GSH-Px reduces both H_2_O_2_ and lipid hydroperoxides including t-BHP–derived metabolites to their corresponding alcohols, thereby protecting cellular membranes from oxidative damage. Treatment with t-BHP significantly suppressed intracellular SOD, GSH-Px, and CAT activities [36]. This inhibition was significantly reversed, and the activities of all three enzymes were effectively preserved following flavonoid pretreatment. These findings indicate that FDF confers broad-spectrum protection not limited to a single oxidant but rather enhances the integrated cellular antioxidant defense network. Thus, *Dendrobium flexicaule* flavonoids alleviate t-BHP–induced oxidative damage in HepG2 cells, at least in part, by preserving the activity of key antioxidant enzymes (SOD, GSH-Px, and CAT), thereby augmenting the cell’s capacity to scavenge peroxides and reactive oxygen species[4]. Collectively, these results demonstrate that FDF alleviates t-BHP–induced oxidative damage in HepG2 cells by modulating the activity of key antioxidant enzymes thereby enhancing the functional capacity of the cellular antioxidant defense system.

### 3.7 FDF upregulates the expression of HO-1 and NQO1 by activating the Nrf2 signaling pathway

**Fig. 8:**
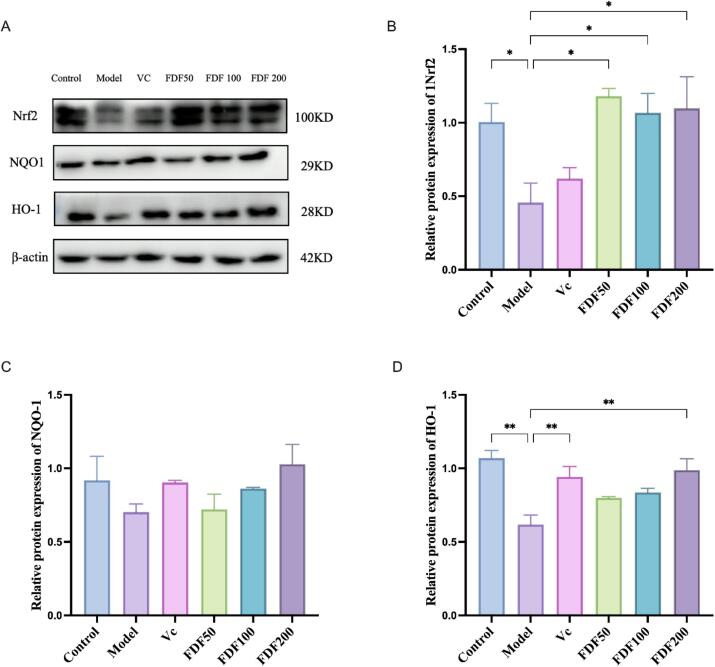
Effects of different concentrations of FDF on the Nrf2/HO-1 antioxidant signaling pathway in HepG2 cells.

As shown in Fig. 8, t-BHP exposure induced significant suppression of the Nrf2/ARE antioxidant defense axis in HepG2 cells, evidenced by marked downregulation of nuclear Nrf2 protein levels, along with corresponding reductions in cytoprotective downstream effectors HO-1 and NQO1. In contrast, pretreatment with ascorbic acid or the optimized *Dendrobium flexicaule* flavonoid extract for 2 h prior to t-BHP challenge fully restored nuclear Nrf2 translocation and significantly upregulated HO-1 and NQO1 expression relative to the t-BHP–treated model group, with FDF exhibiting comparable efficacy to VC. These results demonstrate that the optimized *Dendrobium flexicaule* flavonoid extract (FDF) enhances the antioxidant capacity of HepG2 cells by promoting Nrf2 nuclear translocation, thereby activating the Nrf2/ARE signaling pathway and upregulating the expression of HO-1 and NQO1. The Nrf2/ARE pathway serves as the master transcriptional regulator of the cellular antioxidant defense system[37]. Under oxidative stress, Nrf2 dissociates from its cytosolic inhibitor Keap1, translocates to the nucleus, and binds to antioxidant response elements (AREs) in the promoter regions of target genes thereby initiating transcription of phase II detoxification enzymes, including heme oxygenase-1 (HO-1) and NAD(P)H quinone dehydrogenase 1 (NQO1)[38]. Our findings are consistent with prior reports demonstrating that natural compounds mitigate t-BHP–induced oxidative damage in HepG2 cells via Nrf2 pathway activation. For example, luteolin has been shown to attenuate t-BHP–induced oxidative stress in HepG2 cells by promoting Nrf2 nuclear translocation and upregulating HO-1 expression[39]. Similarly, methyl isoeugenol has been reported to promote Nrf2 nuclear translocation and upregulate the expression of HO-1 and NQO1, thereby conferring protection against oxidative injury [40]. In summary, this study demonstrates that the optimized *Dendrobium flexicaule* flavonoid extract (FDF) significantly enhances the antioxidant capacity of HepG2 cells by activating the Nrf2/ARE signaling pathway thereby upregulating HO-1 and NQO1 expression and strengthening cellular antioxidant activity.

## 4 Conclusion

This study systematically identified *Dendrobium flexicaule* as a flavonoid-rich resource among four *Dendrobium* species. Extraction process optimization was achieved through an integrated modeling framework that synergistically combined response surface methodology (RSM) with machine learning–based predictive modeling; among the evaluated algorithms were support vector regression (SVR), random forest (RF), and artificial neural network (ANN). SVR exhibited the highest predictive accuracy and superior generalizability across independent validation sets, outperforming the conventional quadratic RSM model. Targeted metabolomic profiling identified 34 structurally characterized flavonoid compounds including flavones, flavonols, and flavanones in the optimized D. flexicaule extract. Functional validation confirmed that this flavonoid-enriched extract exerted concentration-dependent antioxidant effects in human hepatocellular carcinoma (HepG2) cells under *tert*-butyl hydroperoxide (t-BHP)–induced oxidative stress: it significantly attenuated intracellular ROS accumulation, restored glutathione (GSH) levels, and activated the Nrf2/ARE signaling axis, as demonstrated by nuclear translocation of Nrf2 and upregulated protein expression of downstream effectors HO-1 and NQO1. These findings establish a robust, integrative methodological framework that synergistically couples targeted metabolomic profiling with machine learning-driven process optimization, demonstrating broad applicability for rational development of plant-derived bioactive ingredients. Critically, the optimized *Dendrobium flexicaule* flavonoid extract (FDF) exhibits not only high compositional fidelity (34 structurally confirmed flavonoids) and reproducible extraction efficiency but also mechanistically validated Nrf2-mediated antioxidant activity in human cells, thereby positioning FDF as a scientifically substantiated candidate for functional food formulation and pharmaceutical-grade nutraceutical development.

### 4.1 Research limitation

(1) All *Dendrobium flexicaule* samples were sourced from a single geographical origin (Chongqing, China), without accounting for agro-climatic variability, harvest seasonality, or intra-species genetic diversity factors known to significantly modulate flavonoid biosynthesis and accumulation in medicinal orchids. Consequently, the empirically optimized extraction parameters may require recalibration for materials harvested from distinct ecological niches.

(2) Antioxidant efficacy was assessed exclusively in an in vitro HepG2 cellular model under t-BHP–induced oxidative stress; while this system robustly elucidated the Nrf2/ARE activation mechanism, translational relevance necessitates validation in physiologically relevant in vivo models, including pharmacokinetic profiling, chronic toxicity assessment, and disease-modifying endpoints in rodent oxidative stress models before clinical evaluation.

### 4.2 Future scope

Future work will therefore prioritize multi-regional sampling across major D. flexicaule cultivation zones (Sichuan, Guizhou, Yunnan) and initiate preclinical in vivo studies to bridge the mechanistic findings with therapeutic applicability.

## CRediT authorship contribution statement

**Liu Yang:** Writing – original draft, Investigation, Formal analysis, Data curation, Conceptualization. **Yuhang Yi:** Writing – review & editing. **Abdulaziz Nuhu Jibril:** Writing – review & editing. **Jing Wen:** Validation, Formal analysis. **Xing Song:** Validation, Formal analysis. **Chenghao Lv:** Methodology, Funding acquisition, Conceptualization. **Si Qin:** Writing – review & editing, Resources, Funding acquisition, Conceptualization.

## Funding

This work was partially supported by National Key Research and Development Program of China (2019YFC1604903) and Natural Science Foundation of Hunan Province (2023JJ30295), Research Project of Hunan Agricultural University (xczx-2025827) to Si Qin.

## Declaration of competing interest

The authors declare that they have no known competing financial interests or personal relationships that could have appeared to influence the work reported in this paper.

## Appendix A Supplementary data

Supplementary Data 1 Metabolomics data.

## References

[1] Wang F., Yuan C., Deng R., Liu Y. (2025). Multi-omics analysis reveals the pre-protective mechanism of Dendrobium flexicaule polysaccharide against alcohol-induced gastric mucosal injury. Int. J. Biol. Macromol..

[2] Li Z., Li J., Shu Z., Xu M., Zhang Y., Gu J., Chen J., Li X., Wang M. (2025). Comparative metabolomic analysis provides insights into the metabolite profiles of wild and cultivated Dendrobium flexicaule. BMC Plant Biol..

[3] Sies H. (2015). Oxidative stress: a concept in redox biology and medicine. Redox Biol..

[4] Zhu P., Wang X., Liu X., Shen X., Li A., Zheng X., Sheng J., Yuan W. (2024). Characterization of the composition of bioactive fractions from dendrobium officinale flowers that protect against H2O2-induced oxidative damage through the PI3K/AKT/Nrf2 pathway. Foods.

[5] Yuan Y., Zuo J., Wan X., Zhou R., Xing W., Liu S. (2024). Multi-omics profiling reveal responses of three major dendrobium species from different growth years to medicinal components. Front. Plant Sci..

[6] Panche A.N., Diwan A.D., Chandra S.R. (2016). Flavonoids: an overview. J. Nutr. Sci..

[7] Zhou Q., Zhang N., Hu T., Xu H., Duan X., Liu B., Chen F., Wang M. (2022). Dietary phenolic-type Nrf2-activators: implications in the control of toxin-induced hepatic disorders. Food Funct..

[8] Bas T.G. (2026). Dietary polyphenols (flavonoids) derived from plants for use in therapeutic health: antioxidant performance. ROS, Molecular Mechanisms, and Bioavailability Limitations, Int J Mol Sci.

[9] Li R., Wang Z., Kong K.W., Xiang P., He X., Zhang X. (2022). Probiotic fermentation improves the bioactivities and bioaccessibility of polyphenols in dendrobium officinale under in vitro simulated gastrointestinal digestion and fecal fermentation. Front. Nutr..

[10] Ren B., Wang M., Hao D., Wang Z., Dai L. (2025). Dendrobium officinale extract alleviates aging-induced kidney injury by inhibiting oxidative stress via the PI3K/akt/Nrf2/HO-1 pathway. J. Ethnopharmacol..

[11] Zuo Y., Jibril A.N., Yan J., Xia Y., Liu R., Chen K. (2025). Optimization of online moisture prediction model for paddy in low-temperature circulating heat pump drying system with artificial neural network. Sens.

[12] El-Mesery H.S., Jibril A.N., ElMesiry A.H., Hu Z., Zhang X., Mahdi A.A. (2025). Artificial neural network and machine learning predictive model for assessing physicochemical properties of garlic slices (allium sativum L.) during microwave-assisted convective drying process. Food Chem.: X.

[13] Subramani V., Tomer V., Balamurali G., Mansingh P. (2025). Artificial neural network in optimization of bioactive compound extraction: recent trends and performance comparison with response surface methodology. Anal. Sci.: Int. J. Jpn. Soc. Anal. Chem..

[14] Mungwari C.P., Obadele B.A., King’ondu C.K. (2025). Application of response surface methodology (RSM) and artificial neural network (ANN) for bioactive compounds recovery from mimosa wattle tree *(acacia mearnsii)* bark using ultrasound-assisted extraction. Sci. Afr..

[15] Jiang D.-Z., Yu D.-P., Zeng M., Liu W.-B., Li D.-L., Liu K.-Y. (2024). Optimization of ultrasonic-assisted extraction of total flavonoids from Oxalis corniculata by a hybrid response surface methodology-artificial neural network-genetic algorithm (RSM-ANN-GA) approach, coupled with an assessment of antioxidant activities. RSC Adv..

[16] Ning S., Honglin D., Xue W., Zengyang H.E., Yuting J., Wei H.a.N., Guokai W., Peng Z.O.U. (2021). Optimization of green extraction process of total flavonoids from *dendrobium officinale* flower. Sci. Technol. Food Ind..

[17] ShuoHAN, Cheng-wangLI, Li-meiWANG, PengGONG, Zeng-huiLIU, Optimization of extraction and purification process of *dendrobium huoshanense* C.Z.tang et S.J.cheng leaf flavonoids and its component analysis, Nat. Prod. Res. Dev. 37 (2025) 95–103. doi: 10.16333/j.1001-6880.2025.1.011.

[18] Jun W.U., Junting W.U., Biwen Y., Mei W., Pei Z., Jingqiu M.A., Yue H., Chuanshu H. (2024). Optimization of ultrasonic-enzyme-assisted deep eutectic solvents extraction process of total flavonoids from mulberry leaves and its antioxidant activity. Sci. Technol. Food Ind..

[19] Xie Y., Guo Q.-S., Wang G.-S. (2016). Preparative separation and purification of the total flavonoids in scorzonera austriaca with macroporous resins. Mol. (basel Switz.).

[20] Lu H., Yang K., Zhan L., Lu T., Chen X., Cai X., Zhou C., Li H., Qian L., Lv G., Chen S. (2019). Optimization of flavonoid extraction in dendrobium officinale leaves and their inhibitory effects on tyrosinase activity. Int. J. Anal. Chem..

[21] Wu J., He T., Wang Z., Mao J., Sha R. (2024). The dynamic analysis of non-targeted metabolomics and antioxidant activity of dendrobium officinale kimura et migo by the synergistic fermentation of bacteria and enzymes. LWT.

[22] Johnston J.W., Dussert S., Gale S., Nadarajan J., Harding K., Benson E.E. (2006). Optimisation of the azinobis-3-ethyl-benzothiazoline-6-sulphonic acid radical scavenging assay for physiological studies of total antioxidant activity in woody plant germplasm. Plant Physiol. Biochem..

[23] Lin T., Ye Y., Zhang J., Wang J., Hu Z., Linn K.Z., Chen X., Liu H., Liu Z., Yao Q. (2025). Machine Learning and UHPLC-MS/MS-Based Discrimination of the Geographical Origin of Dendrobium officinale from Yunnan. China, Foods.

[24] Deng Y., Li S., Shi Y.-R., Hu D.-B., Luo J.-F., Zhao P.-J., Yuan W.-J., Wang Y.-H. (2024). Variation in the contents of four flavonoid glycosides in edible Dendrobium officinale leaves during different harvesting periods and optimization of the extraction process. Food Chem.: X.

[25] Yang Y., Guan Y., Li S., Xu Y. (2025). Bioactive Ingredient Profiling of Dendrobium officinale: Plant-Part-specific distribution of Key Metabolites and their Multi-Disease Therapeutic potential. Metabolites.

[26] Yuan Y., Zuo J., Zhang H., Li R., Yu M., Liu S. (2022). Integration of Transcriptome and Metabolome Provides New Insights to Flavonoids Biosynthesis in Dendrobium huoshanense. Front. Plant Sci..

[27] Chen S., Zhang F., da Silva A.P.G., Simal-Gandara J., Cao H. (2025). Vitamin C prevents myricetin degradation in boiling water by reducing ortho-quinone intermediates. Food Chem..

[28] Qiao L., Sun Y., Chen R., Fu Y., Zhang W., Li X., Chen J., Shen Y., Ye X. (2014). Sonochemical effects on 14 flavonoids common in citrus: relation to stability. PLoS One.

[29] Huaman-Castilla N.L., Martínez-Cifuentes M., Camilo C., Pedreschi F., Mariotti-Celis M., Pérez-Correa J.R. (2019). The Impact of Temperature and Ethanol Concentration on the Global Recovery of specific Polyphenols in an Integrated HPLE/RP Process on Carménère Pomace Extracts. Molecules.

[30] Zhang H., Xie G., Tian M., Pu Q., Qin M. (2016). Optimization of the ultrasonic-assisted extraction of bioactive flavonoids from ampelopsis grossedentata and subsequent separation and purification of two flavonoid aglycones by high-speed counter-current chromatography. Molecules.

[31] Wu T.-W., Chu Y.-C., Chang C.-H., Hsieh Y.-H., Tang M.-H., Hsu P.-H., Wu H.-Y., Chen J.-J., Shih T.-L. (2024). Flavonol-Ruthenium Complexes as Antioxidant and Anticancer Agents. ChemMedChem.

[32] Cui Y., Xiong Y., Li H., Zeng M., Wang Y., Li Y., Zou X., Lv W., Gao J., Cao R., Meng L., Long J., Liu J., Feng Z. (2021). Chalcone-Derived Nrf2 Activator Protects Cognitive Function via Maintaining Neuronal Redox Status. Antioxidants (basel).

[33] Hu J.P., Calomme M., Lasure A., De Bruyne T., Pieters L., Vlietinck A., D.a. (1995). Vanden Berghe, Structure-activity relationship of flavonoids with superoxide scavenging activity. Biol. Trace Elem. Res..

[34] Yang J., Xue Q., Li C., Jin Y., Xue Q., Liu W., Niu Z., Ding X. (2025). A chromosome-level Dendrobium moniliforme genome assembly reveals the regulatory mechanisms of flavonoid and carotenoid biosynthesis pathways. Acta Pharm. Sin. B.

[35] Pizzimenti S., Ciamporcero E., Daga M., Pettazzoni P., Arcaro A., Cetrangolo G., Minelli R., Dianzani C., Lepore A., Gentile F., Barrera G. (2013). Interaction of aldehydes derived from lipid peroxidation and membrane proteins. Front. Physiol..

[36] Shivananjappa M.M., Mhasavade D., Joshi M.K. (2013). Aqueous extract of terminalia arjuna attenuates tert-butyl hydroperoxide-induced oxidative stress in HepG2 cell model. Cell Biochem. Funct..

[37] Huang W., Zhong Y., Gao B., Zheng B., Liu Y. (2023). Nrf2-mediated therapeutic effects of dietary flavones in different diseases. Front. Pharmacol..

[38] Kobayashi A., Ohta T., Yamamoto M. (2004). Unique function of the Nrf2-Keap1 pathway in the inducible expression of antioxidant and detoxifying enzymes. Methods Enzymol..

[39] Kitakaze T., Makiyama A., Samukawa Y., Jiang S., Yamashita Y., Ashida H. (2019). A physiological concentration of luteolin induces phase II drug-metabolizing enzymes through the ERK1/2 signaling pathway in HepG2 cells. Arch. Biochem. Biophys..

[40] Liu H., Chen W., He M., Nie L., Pan Y., Guan D., Li Y., Wan T., Duan L., Yang C., Li W., Wang Q., Zhuang L., Zhang Y. (2025). Methyl isoeugenol suppresses NLRP3 inflammasome-mediated pyroptosis via activation of Nrf2/NQO1/HO-1 signaling in cerebral ischemia-reperfusion injury. Biochem. Pharmacol..

