# Supplementary material for: Optimization of flavonoids extraction and elucidation of antioxidant mechanisms in Dendrobium flexicaule using metabolomics and machine learning
Source: Ultrason Sonochem. 2026 Jul 5;131:107950. doi: 10.1016/j.ultsonch.2026.107950 (PMC13351277; doi:10.1016/j.ultsonch.2026.107950)
Supplement: Supplementary Data 1 — Metabolomics data. [file mmc1.docx]

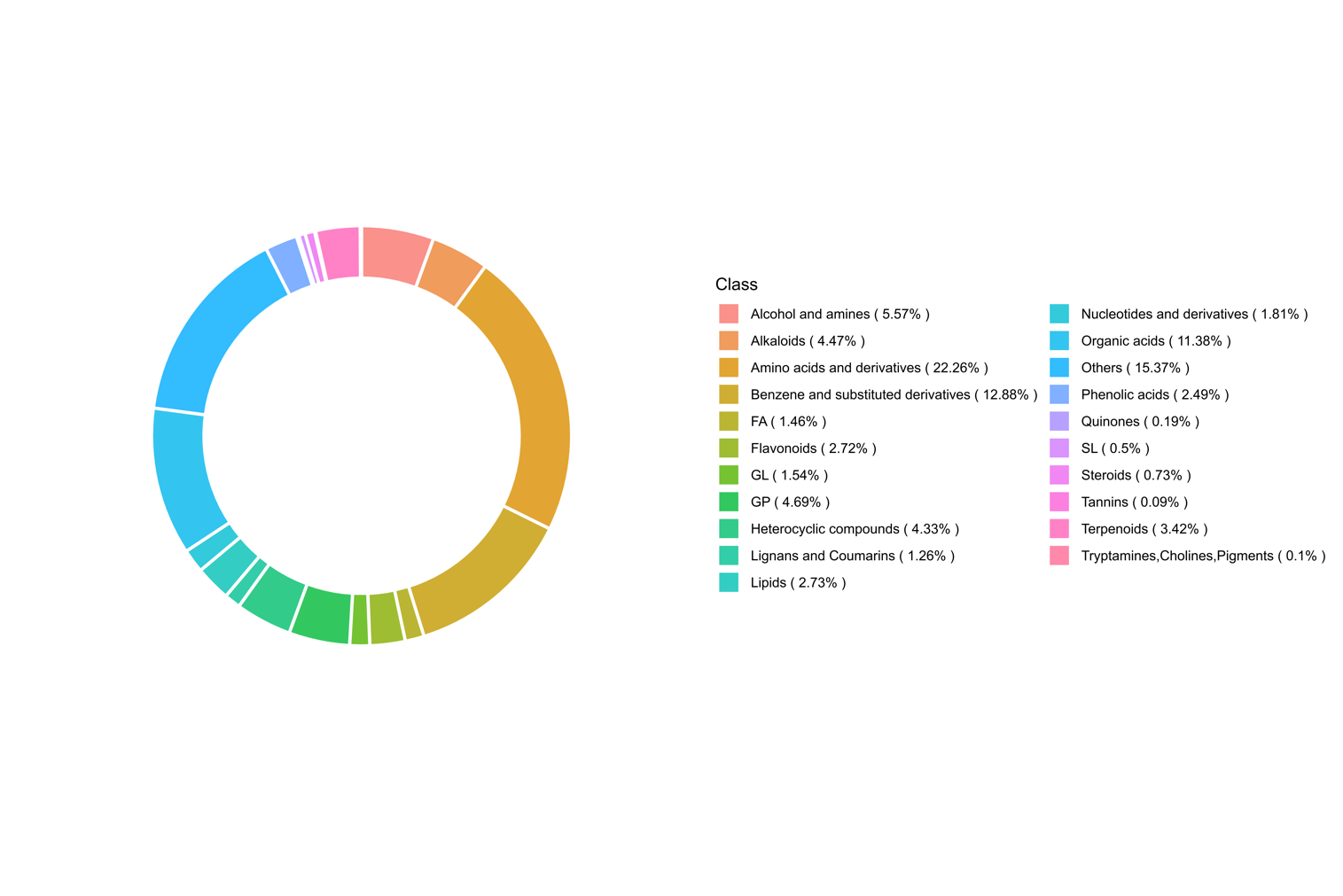


Supplementary Figure S1: Alcohol extract of *Dendrobium flexicaule*


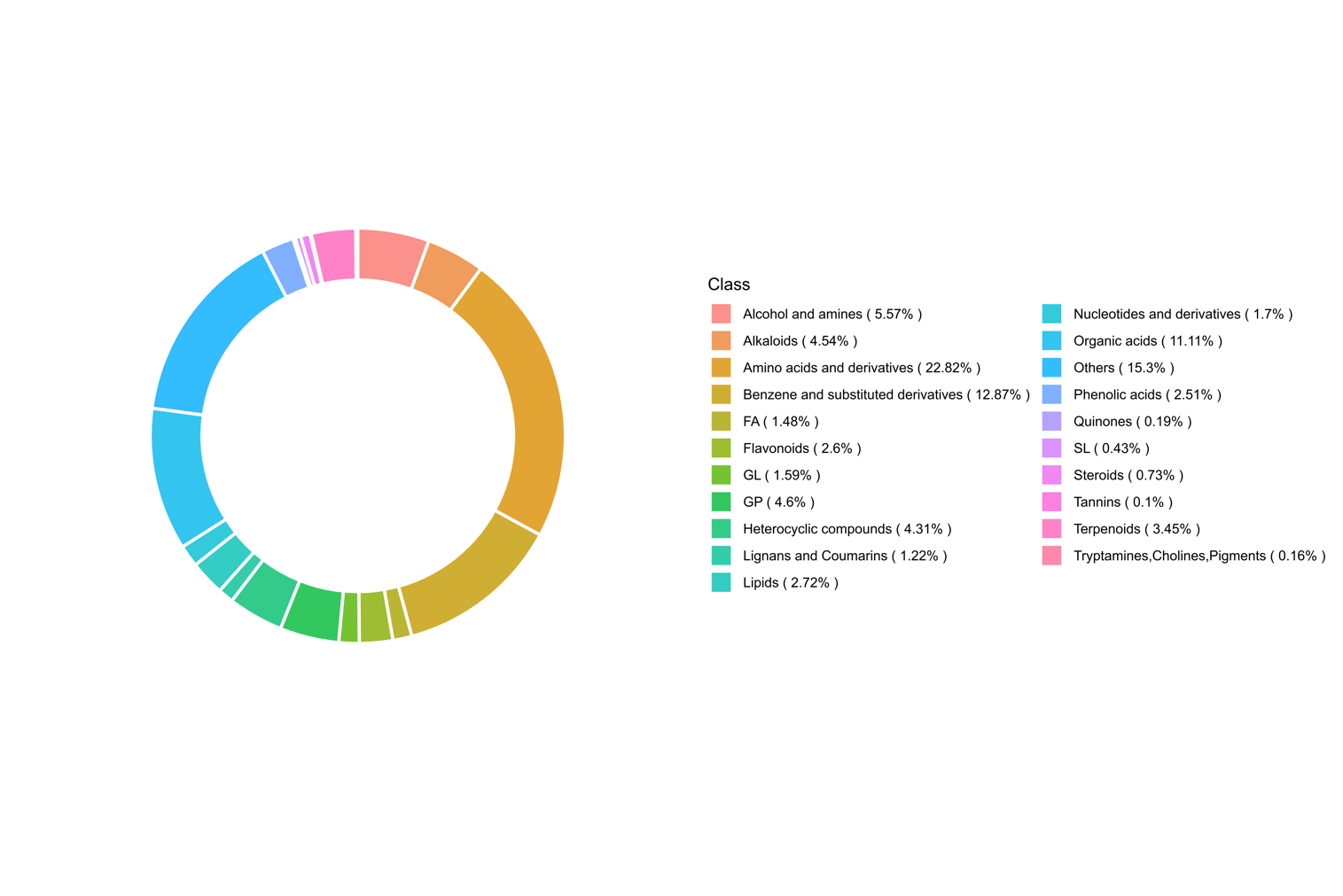


Supplementary Figure S2: Aqueous extract of *Dendrobium flexicaule*


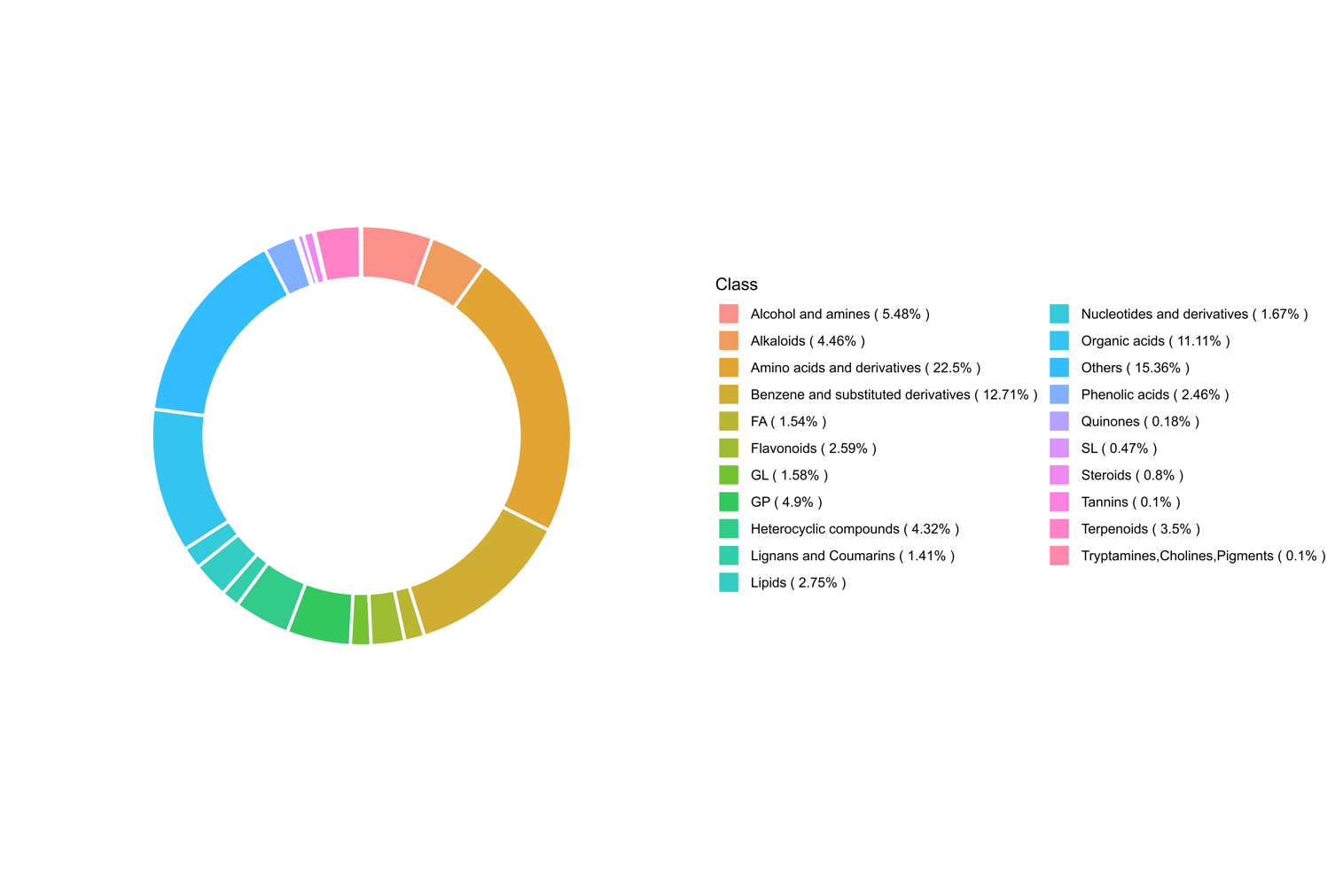


Supplementary Figure S3: Alcohol extract of *Dendrobium huoshanense*


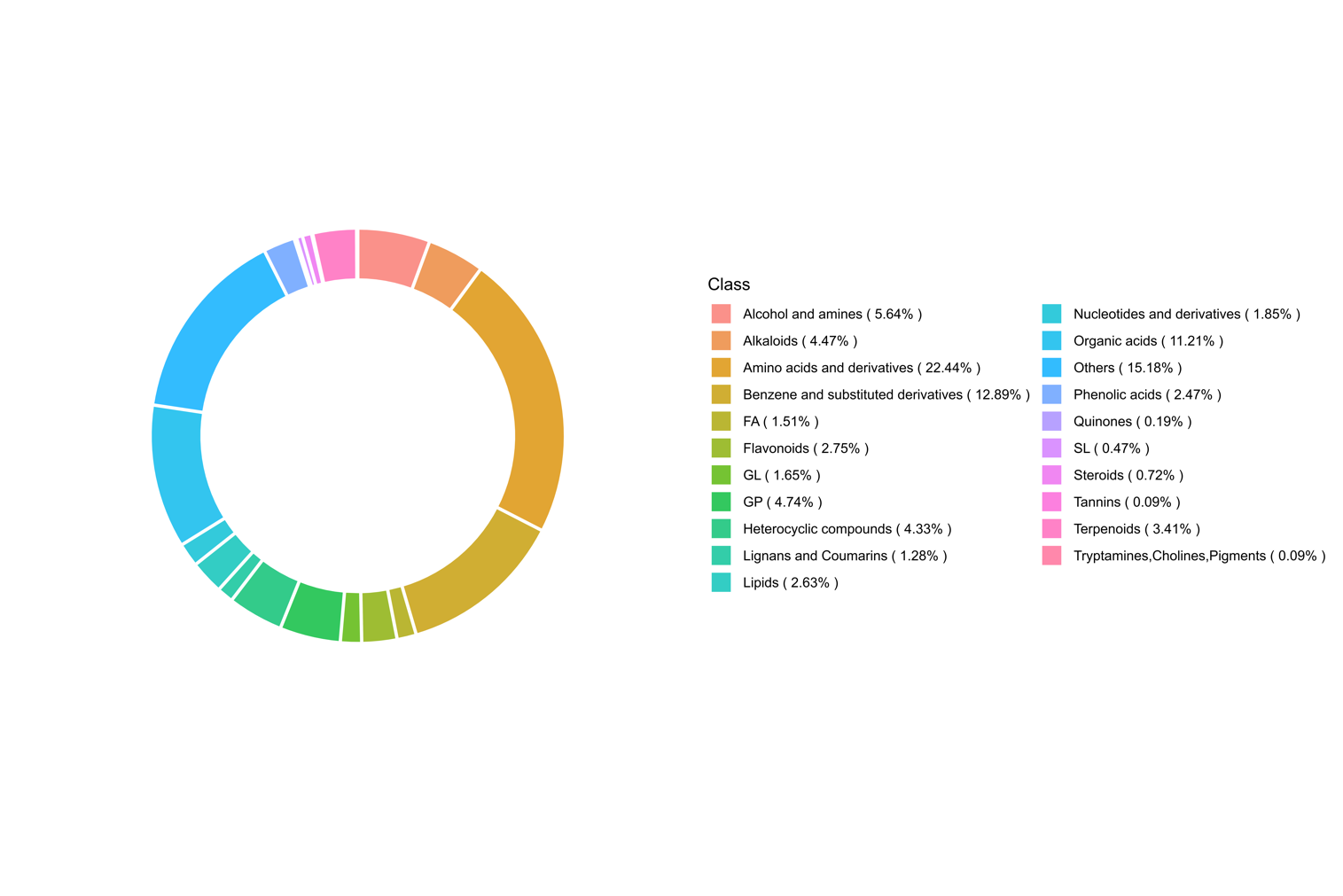


Supplementary Figure S4: Aqueous extract of *Dendrobium huoshanense*


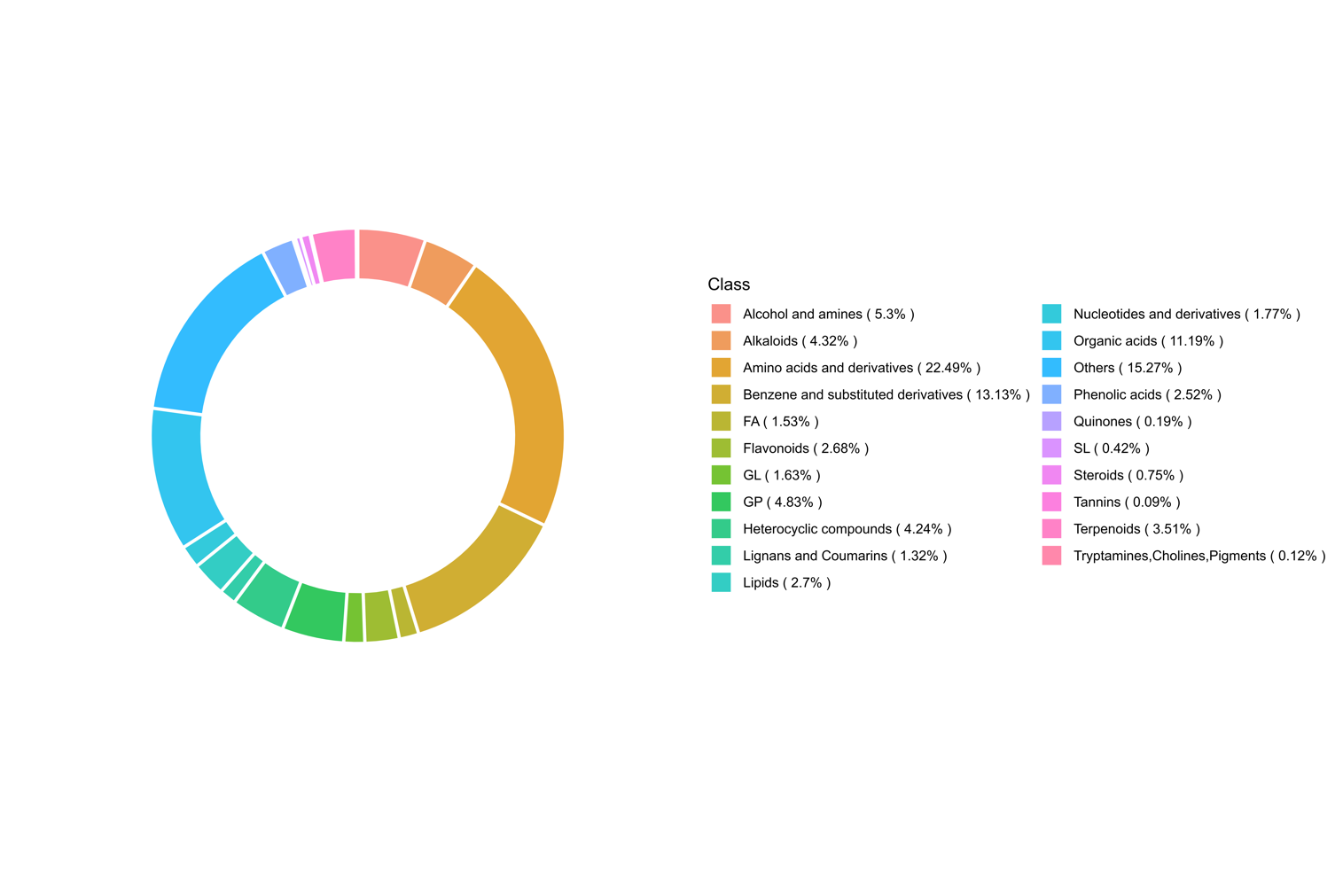


Supplementary Figure S5: Alcohol extract of *Dendrobium officinale* (3-year)


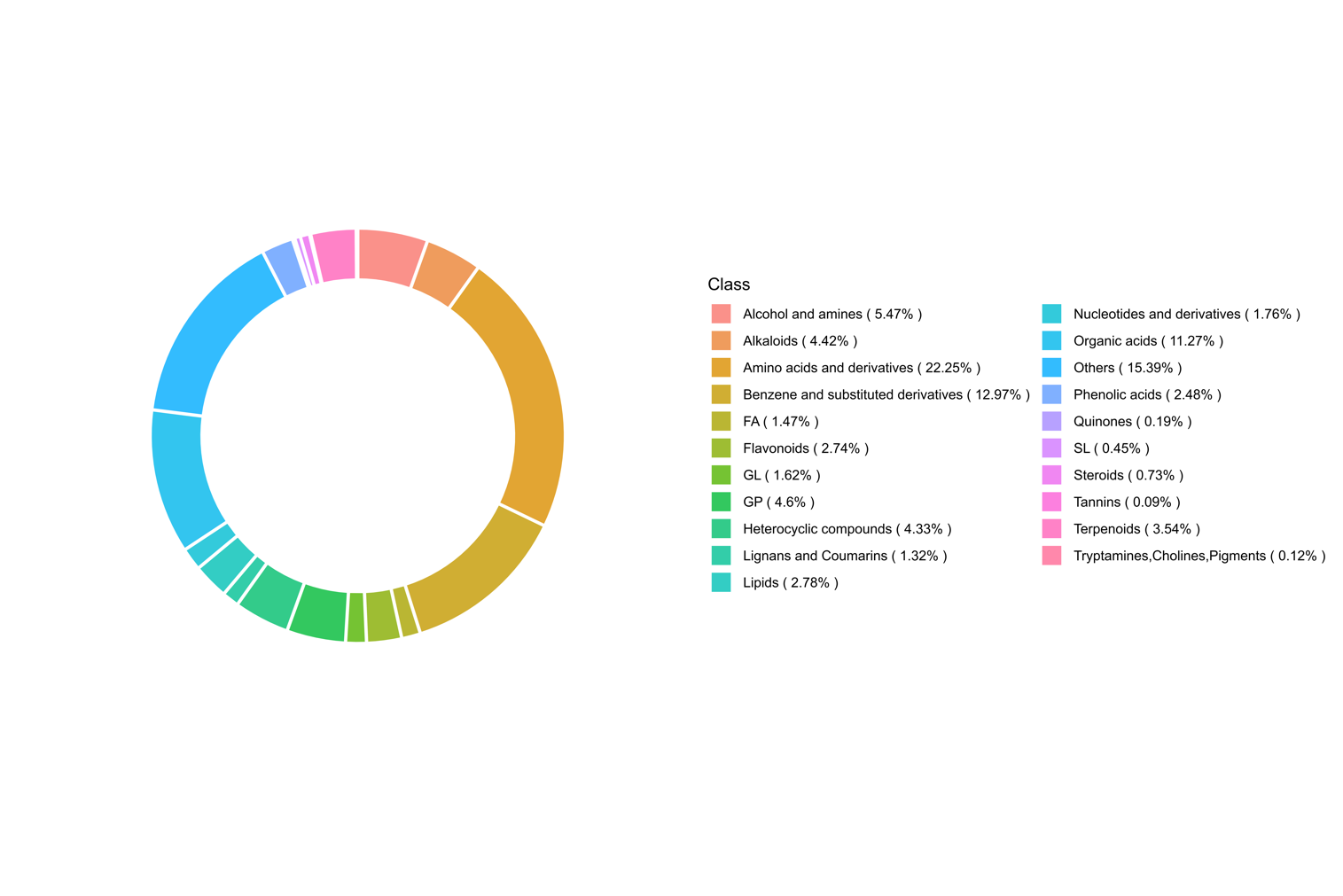


Supplementary Figure S6: Aqueous extract of *Dendrobium officinale* (3-year)


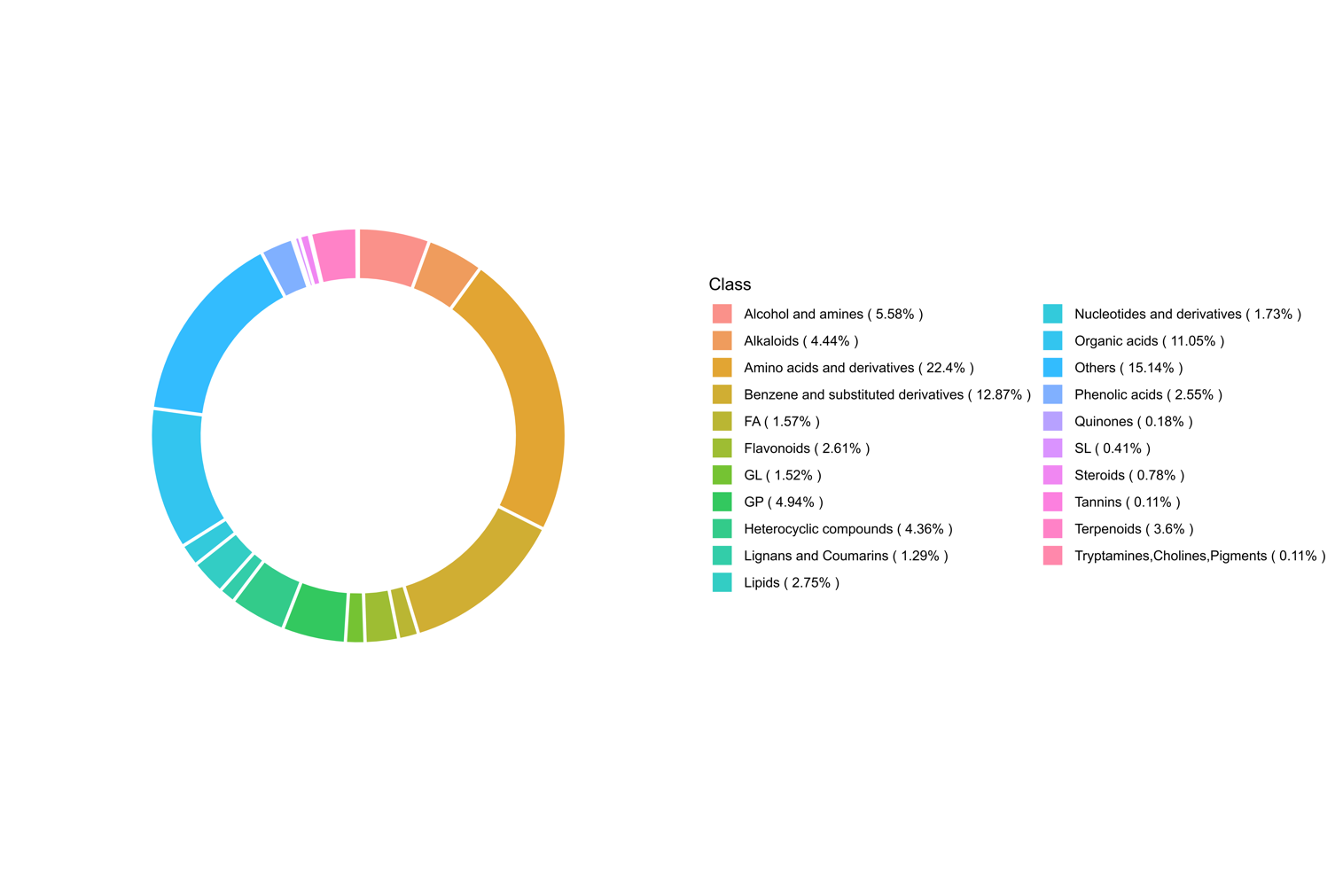


Supplementary Figure S7: Alcohol extract of *Dendrobium officinale* (5-year)


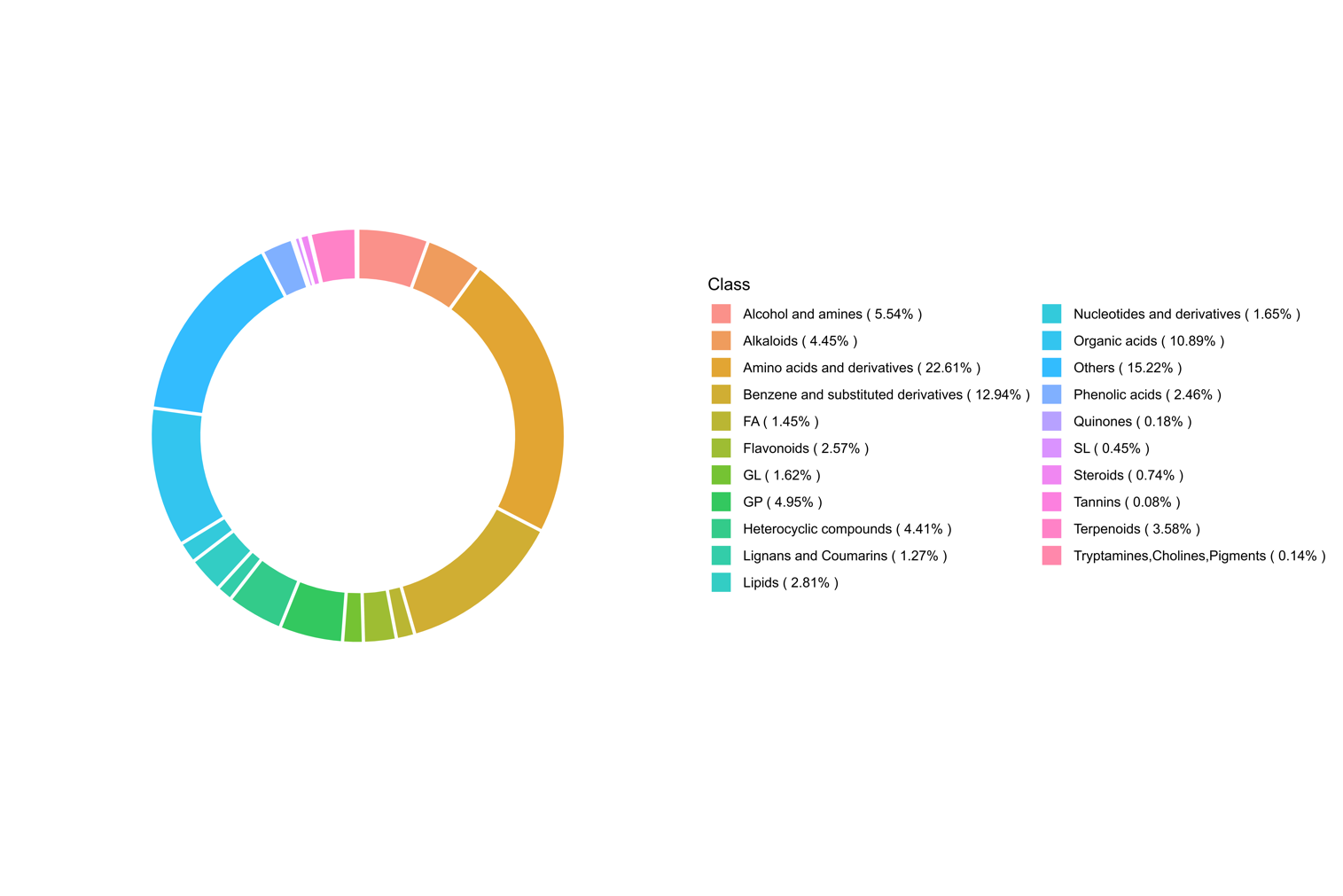


Supplementary Figure S8: Aqueous extract of Dendrobium officinale (5-year)


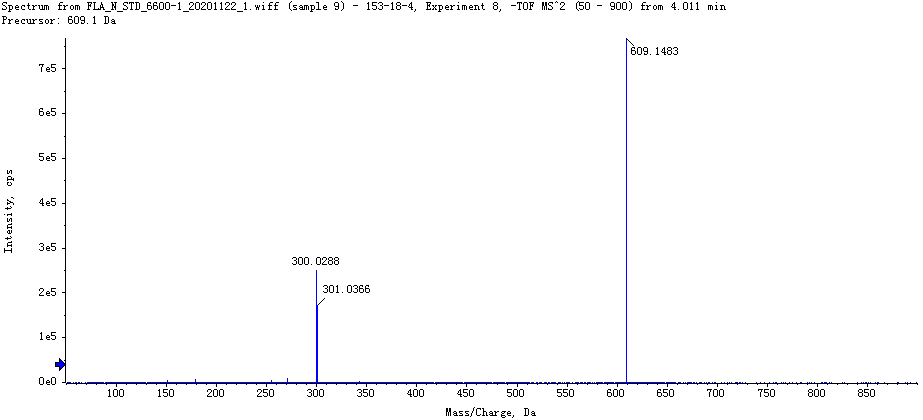


Supplementary Figure S9**:** Representative MS/MS spectrum of rutin.


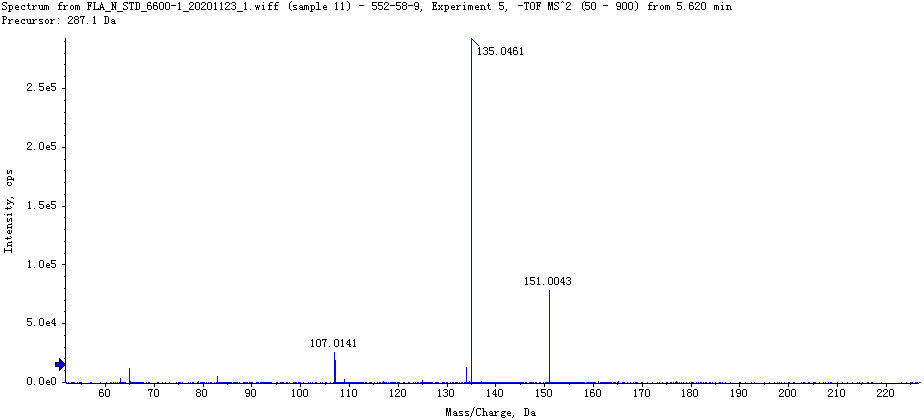


Supplementary Figure S10. Representative MS/MS spectrum of eriodictyol.


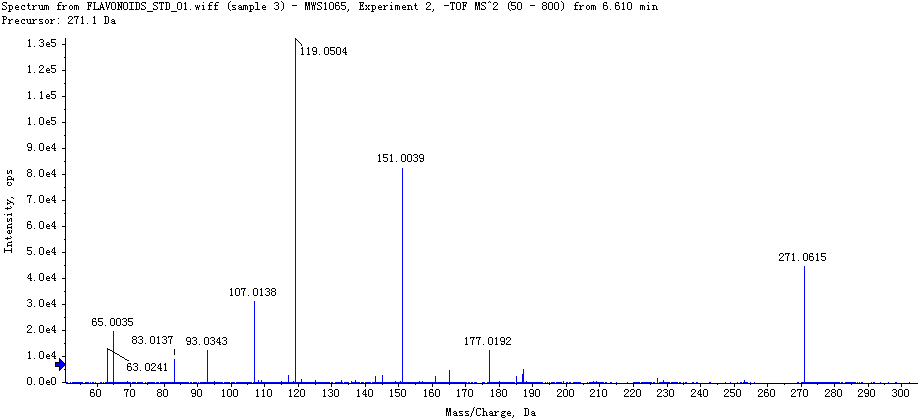


Supplementary Figure S10. Representative MS/MS spectrum of eriodictyol.

**Supplementary Table S1. Detailed information of 34 authentic standards used for MSI Level 1**

|  | Index | Compounds | Molecular Weight | Formula | CAS | RT | MSI Level |
| --- | --- | --- | --- | --- | --- | --- | --- |
| 1 | Flavonoid_138 | Baimaside | 626.148305 | C_27_H_30_O_17_ | 18609-17-1 | 2.34 | **Level 1** |
| 2 | Flavonoid_42 | Nicotiflorin | 594.158475 | C_27_H_30_O_15_ | 17650-84-9 | 2.74 | **Level 1** |
| 3 | Flavonoid_139 | Dihydrokaempferol | 288.06339 | C_15_H_12_O_6_ | 480-20-6 | 3.41 | **Level 1** |
| 4 | Flavonoid_72 | Apigenin | 270.052825 | C_15_H_10_O_5_ | 520-36-5 | 4.66 | **Level 1** |
| 5 | Flavonoid_191 | Naringenin chalcone | 272.068475 | C_15_H_12_O_5_ | 25515-46-2 | 4.65 | **Level 1** |
| 6 | Flavonoid_177 | Phloretin | 274.084125 | C_15_H_14_O_5_ | 60-82-2 | 4.58 | **Level 1** |
| 7 | Flavonoid_118 | Narcissin | 624.16904 | C_28_H_32_O_16_ | 604-80-8 | 2.78 | **Level 1** |
| 8 | Flavonoid_06 | Diosmetin | 300.06339 | C_16_H_12_O_6_ | 520-34-3 | 4.91 | **Level 1** |
| 9 | Flavonoid_154 | Vitexin | 432.10565 | C_21_H_20_O_10_ | 3681-93-4 | 2.64 | **Level 1** |
| 10 | Flavonoid_58 | Isorhamnetin | 316.058305 | C_16_H_12_O_7_ | 480-19-3 | 4.99 | **Level 1** |
| 11 | Flavonoid_197 | Isorhamnetin 3-O-glucoside | 478.11113 | C_22_H_22_O_12_ | 5041-82-7 | 2.92 | **Level 1** |
| 12 | Flavonoid_126 | Eriodictyol | 288.06339 | C_15_H_12_O_6_ | 552-58-9 | 3.90 | **Level 1** |
| 13 | Flavonoid_57 | Rutin | 610.15339 | C_27_H_30_O_16_ | 153-18-4 | 2.57 | **Level 1** |
| 14 | Flavonoid_108 | Pinocembrin | 256.07356 | C_15_H_12_O_4_ | 480-39-7 | 6.84 | **Level 1** |
| 15 | Flavonoid_56 | Taxifolin | 304.058305 | C_15_H_12_O_7_ | 480-18-2 | 2.96 | **Level 1** |
| 16 | Flavonoid_146 | Icariin | 676.236725 | C_33_H_40_O_15_ | 489-32-7 | 3.86 | **Level 1** |
| 17 | Flavonoid_23 | Quercetin | 302.042655 | C_15_H_10_O_7_ | 117-39-5 | 3.98 | **Level 1** |
| 18 | Flavonoid_186 | Prunetin | 284.068475 | C_16_H_12_O_5_ | 552-59-0 | 7.01 | **Level 1** |
| 19 | Flavonoid_50 | Luteolin | 286.04774 | C_15_H_10_O_6_ | 491-70-3 | 3.94 | **Level 1** |
| 20 | Flavonoid_09 | Genistein | 270.052825 | C_15_H_10_O_5_ | 446-72-0 | 4.72 | **Level 1** |
| 21 | Flavonoid_201 | Trilobatin | 436.13695 | C_21_H_24_O_10_ | 4192-90-9 | 3.35 | **Level 1** |
| 22 | Flavonoid_119 | Astragalin | 448.100565 | C_21_H_20_O_11_ | 480-10-4 | 2.89 | **Level 1** |
| 23 | Flavonoid_86 | Avicularin | 434.084915 | C_20_H_18_O_11_ | 572-30-5 | 2.89 | **Level 1** |
| 24 | Flavonoid_35 | Syringaldehyde | 182.05791 | C_9_H_10_O_4_ | 134-96-3 | 2.99 | **Level 1** |
| 25 | Flavonoid_198 | 5,7-Dihydroxy-3',4',5'-trimethoxyflavone | 344.089605 | C_18_H_16_O_7_ | 18103-42-9 | 6.47 | **Level 1** |
| 26 | Flavonoid_171 | 6-Hydroxyflavone | 238.062995 | C_15_H_10_O_3_ | 6665-83-4 | 5.60 | **Level 1** |
| 27 | Flavonoid_165 | Homoplantaginin | 462.116215 | C_22_H_22_O_11_ | 17680-84-1 | 2.99 | **Level 1** |
| 28 | Flavonoid_158 | Isosakuranetin | 286.084125 | C_16_H_14_O_5_ | 480-43-3 | 6.76 | **Level 1** |
| 29 | Flavonoid_43 | Kaempferol | 286.04774 | C_15_H_10_O_6_ | 520-18-3 | 4.81 | **Level 1** |
| 30 | Flavonoid_98 | Hyperoside | 464.09548 | C_21_H_20_O_12_ | 482-36-0 | 2.64 | **Level 1** |
| 31 | Flavonoid_61 | Chrysin | 254.05791 | C_15_H_10_O_4_ | 480-40-0 | 6.66 | **Level 1** |
| 32 | Flavonoid_30 | Isoliquiritigenin | 256.07356 | C_15_H_12_O_4_ | 961-29-5 | 5.38 | **Level 1** |
| 33 | Flavonoid_160 | Apigenin 7-glucoside | 432.10565 | C_21_H_20_O_10_ | 578-74-5 | 2.96 | **Level 1** |
| 34 | Flavonoid_104 | Corylin | 320.10486 | C_20_H_16_O_4_ | 53947-92-5 | 8.04 | **Level 2** |
